# Large Depth‐of‐Field, Large Eyebox, and Wide Field‐of‐View Freeform‐Holographic Augmented Reality Near‐Eye Display

**DOI:** 10.1002/advs.202508773

**Published:** 2025-07-11

**Authors:** Yongdong Wang, Tong Yang, Xin Lyu, Dewen Cheng, Yongtian Wang

**Affiliations:** ^1^ Beijing Engineering Research Center of Mixed Reality and Advanced Display School of Optics and Photonics Beijing Institute of Technology Beijing 100081 China

**Keywords:** augmented reality, depth of field, freeform optics, holographic optics, near‐eye display

## Abstract

Augmented reality (AR) presents virtual information seamlessly integrated with real‐world scenes, advancing information interaction for human society. Traditional AR displays struggle to simultaneously achieve large depth‐of‐field (DOF), large viewing eyebox, and wide field‐of‐view (FOV), which limits their practical applicability. Here, a novel AR near‐eye display scheme is proposed that addresses these limitations, providing a large DOF, expansive eyebox, and wide FOV. The DOF is largely extended at each viewpoint on the eye pupil plane by directly projecting light rays onto the retina. Meanwhile, numerous viewpoints are generated in space to significantly expand the viewing eyebox, with each viewpoint independently controllable to dynamically track eye movements in real time without the need for mechanical steering parts. Freeform and holographic optics are deeply cooperated in the proposed off‐axis display for achieving high performance over the full FOV, eyebox and DOF range, as well as compact form factor. A display system prototype is built, and the display results, featuring a DOF from 0.25 to 10 m, a 50° wide FOV, and a 10 mm×10 mm viewing eyebox, along with good optical see‐through viewing, demonstrate the effectiveness and advancement of the proposed scheme, offering valuable insights for the future design of AR displays.

## Graphical abstract

This study proposes an augmented reality (AR) near‐eye display system that simultaneously achieves a large depth‐of‐field (DOF) from 0.25 to 10 m, a wide 50° field‐of‐view (FOV), and a 10 mm × 10 mm viewing eyebox, while maintaining high display performance, good optical see‐through viewing capability, and a compact system form factor.

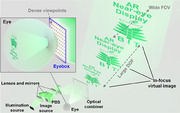

## 1 Introduction

Augmented reality (AR) seamlessly superimposes virtual information onto real‐world scenes, representing a revolutionary advancement in digital information acquisition and interaction, which prompts unprecedented development across various application domains, spanning education, entertainment, healthcare, and industry.^[^ ^1^ ^]^ See‐through near‐eye displays are the core technologies for realizing AR systems, where achieving a large depth‐of‐field (DOF), large viewing eyebox, and wide field‐of‐view (FOV) simultaneously is crucial for ensuring both visual experiences and practical applicability.^[^ ^2^ ^]^ However, great challenges remain in realizing these design requirements at the same time. It is very difficult to realize a large FOV and a large exit‐pupil size owing to the conservations of Étendue in optics as well as the difficulty in aberration correction. The available exit‐pupil size totally determines the viewing eyebox, referring to the spatial region within which the eye can view the complete virtual image. Meanwhile, the DOF range for viewing the virtual image is also traded off against the effective exit‐pupil size across the eye pupil domain.^[^ ^3^ ^]^

**Figure 1:**
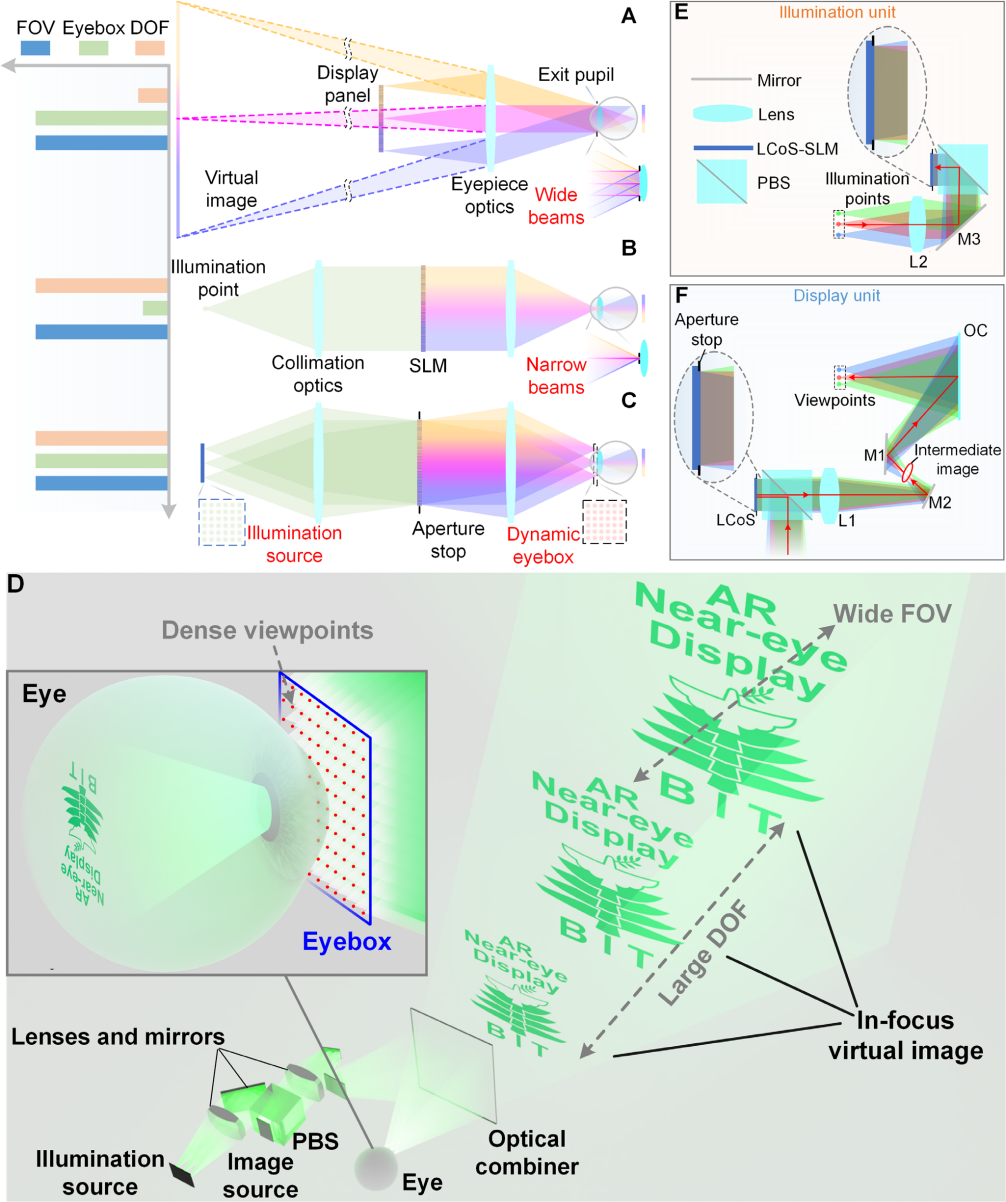
Concept of the proposed scheme. A) Conventional near‐eye displays. B) Extending the DOF by reducing the effective exit‐pupil to a viewpoint at the center of eye pupil. C) Expanding the viewing eyebox by creating a dense viewpoints array, with each individually controllable, while maintaining a large DOF and wide FOV. D) Concept of the proposed large DOF, large eyebox, and wide FOV freeform‐holographic AR near‐eye display. E) Illumination unit. F) Display unit.

Through the optimized collocation of an eyepiece optics and a flat display panel (image source), as shown in **Figure** **1**A, conventional near‐eye displays can optically simulate the waves, which are virtually emitted from the virtual image points located at a specific fixed depth plane in front of eye (the virtual image depth is much larger than the focal length of eyepiece).^[^ ^4^ ^]^ These waves are confined to a fixed area, called as the exit‐pupil, which is equivalent to positioning an aperture stop at the exit‐pupil plane. When the eye pupil is located within the exit‐pupil domain, these waves corresponding to all virtual points are focused by the eye onto the image points on the retina, and the human vision system can perceive the virtual image information. To accommodate the eye movement, the exit‐pupil is required to be larger than the eye pupil size. In general, the pixel units on the image sources (e.g., liquid crystal display (LCD) panel) emit lights over a large solid angle, which offers an adequate Étendue to enable a large exit‐pupil for the system design.^[^ ^5^ ^]^ However, this kind of system shown in Figure 1A imposes a critical limitation on the DOF for viewing the virtual image. The virtual image can only be sharply observed within a limited depth range with the acceptable retina blur, resulting in a fairly limited DOF. This may induce the vergence‐accommodation conflict (VAC), triggering visual fatigue and discomfort. To address this problem, several display schemes have been proposed, including multifocal displays,^[^ ^6^ ^]^ varifocal displays,^[^ ^7^ ^]^ light‐field displays,^[^ ^8^ ^]^ holographic displays;^[^ ^9^ ^]^ however, they still suffer from challenges such as complex and large system structure, small FOV, low resolution, and huge computational loads, etc.

Another effective scheme for extending the DOF in near‐eye displays is to directly reduce the exit‐pupil, as illustrated in Figure 1B, where the exit‐pupil size is smaller than eye pupil, without significantly compromising the FOV and system form factor, which equivalently reduces the display Étendue. To implement this, an amplitude‐type spatial light modulator (SLM) is selected as the image source. A bundle of collimated illumination rays is modulated pixel‐by‐pixel by the SLM,^[^ ^10^ ^]^ essentially creating the image source with a low Étendue here, which results in a dramatic reduction in the effective exit‐pupil. The uniform modulation enabled by the SLM panel requires a collimation beam, which can be simply obtained by using a collimation optics and an illumination point that emits a spherical (or quasi spherical) wave. These narrow light rays loading the image information are focused by the eyepiece optics into an exit‐pupil point, called as the viewpoint here, which is located at the center of eye pupil, and then directly projected onto the retina without focusing by the eye. The above configuration is also known as a Maxwellian display or retinal projection display.^[^ ^11^, ^12^ ^]^ Owing to removing the eye's focus cues, the virtual image is consistently presented sharply on the retina when the eye focuses on real scenes at the different depths, thus extending the DOF range largely. Meanwhile, this display is less susceptible to refractive errors of the eye such as myopia, hyperopia, astigmatism, and presbyopia, facilitating accommodation for a wider range of users without the need for special correction lens.^[^ ^13^ ^]^

However, reducing the effective exit‐pupil to extend the DOF would result in an extremely tiny viewing eyebox. When the eye pupil deviates slightly from the designated viewpoint position, the virtual image will vignette or even disappear entirely. To accommodate eye movements or saccades, the viewing eyebox must be spatially expanded, which essentially requires increasing the display Étendue. Various innovative methods have been proposed in both academia and industry to achieve this, such as statically generating several discrete exit‐pupils within the range of eye movement by utilizing beam splitter (BS) array,^[^ ^14^ ^]^ Pancharatnam‐Berry deflectors (PBD),^[^ ^15^ ^]^ multiplexed holographic optical element (HOE),^[^ ^16^ ^]^ polarization grating (PG),^[^ ^17^ ^]^ Dammann grating (DG),^[^ ^18^, ^19^ ^]^ pin‐mirror HOEs,^[^ ^20^ ^]^ etc. However, these methods generally generate only a limited number of specific and discrete viewpoints, which easily leads to the blind areas or multiple images due to variations in the eye pupil size, and prevents the continuous tracking for the eye pupil positions that can be somewhat random and arbitrary. Although, the exit‐pupil points can be dynamically shifted or deflected in response to the eye saccades by utilizing fast steering mirror (FSM),^[^ ^21^, ^22^ ^]^ moving device,^[^ ^23^ ^]^ polarization controller,^[^ ^24^, ^25^, ^26^ ^]^ light emitting diode (LED) array,^[^ ^27^ ^]^ etc., these typically involve the complicated optomechanical components, resulting in a bulky system volume and form factor, as well as reduced applicability. In addition, a laser scanning projector (LSP) can also be used as the image source; however, expanding the viewing eyebox requires either multiple LSPs or special optical/mechanical devices used with a single LSP,^[^ ^14^, ^15^, ^17^, ^18^, ^21^ ^]^ which would increase the system volume and costs, or result in only a limited number of viewpoints.

Here, we propose a novel scheme to implement an AR near‐eye display with a large DOF and viewing eyebox, as well as wide FOV simultaneously, while realizing a high display performance and compact system form factor. The DOF is largely extended at each viewpoint. The viewing eyebox is expanded by creating a dense and huge amount of viewpoints array in space, where each viewpoint can be independently controlled to dynamically and continuously track the eye pupil positions in real time without employing any mechanical steering or moving devices. Freeform and holographic optics are deeply cooperated in the display to effectively correct aberrations and modulate waves, realizing the desirable performance and see‐through viewing, as well as compact form factor. To validiate the effectiveness and advancement of the proposed scheme, the display system prototype is built, and the display results, featuring a DOF from 0.25 to 10 m, a 50° large FOV, and a 10 mm×10 mm viewing eyebox, as well as good optical see‐through viewing, are realized.

## 2 Proposed AR Near‐Eye Display Scheme

The basic system scheme proposed in this work is shown in Figure 1C. First, in order to extend the DOF, for a single viewpoint, the system structure shown in Figure 1B will be used. However, the Étendue provided by this display is quite limited, resulting in a tiny viewing eyebox. To further create an expanded viewing eyebox range that features an array of individually controllable viewpoints, the Étendue must be increased accordingly. A self‐emissive display panel with high luminance, pixel density and frame rate, such as micro‐LED or organic light emitting diode (OLED) panel is utilized as the illumination source here, where each self‐emissive pixel unit of OLED panel acts as an illumination point mapping to a viewpoint on the eye pupil plane. It is noted that an OLED panel used as the illumination source is intrinsically different from a conventional LED array.^[^ ^27^ ^]^ The pixel size of OLED panel is typically on the micrometer scale. In the case of far field propagation (i.e., when propagation distance is much greater than pixel size), which generally coincides with the practical application, the light wave emitted from a self‐emissive pixel can be approximated as a quasi‐spherical wave perpendicular to the panel surface and can be simply modeled as a point source for the system design. In contrast, a single emissive unit in an LED array has a feature size on the millimeter scale and behaves as an extended source, thereby resulting in a considerably complicated modelling process.^[^ ^28^ ^]^ Additionally, given the size of illumination source, the number of emissive units in the LED array is significantly less than that of OLED panel, which only generates a limited number of viewpoints, leading to reduced applicability. In this way of using an OLED panel as the illumination source, a highly dense viewpoints array can be formed in space, each independently controlled in real time, achieving effective expansion of the display Étendue. As the eye pupil saccades, the viewing eyebox can be dynamically expanded by only lighting the specific illumination points. The mechanical deflection or moving devices are not used, thus greatly enhancing the flexibility and reducing the system complexity.

From the aspect of optical design, the display panel (illumination source) will be in fact the “object” at a finite distance and the eye pupil plane will be the “image plane”. Each “object point” (corresponding to each display pixel) should be stigmatically imaged at the “image plane” in the ideal case in order to achieve large DOF at each viewpoint. The FOV of each viewpoint is equivalent to the “aperture angle” for each “image point” at the “image plane”. Regarding each viewpoint, a complete and consistent image information should be loaded by the SLM. To fully use the pixels on the SLM panel and keep the resolution, the light beams of different viewpoints should be fully overlapped on the plane, which means the SLM plane will be the “aperture stop” in this system. Therefore, the object‐image and pupil change their roles in this system scheme compared to traditional display system scheme as shown in Figure 1A. Under this proposed scheme, the large DOF, large eyebox, and wide FOV AR near‐eye display can be realized, comparing the other schemes. It is noted that the Figure 1C only depicts the schematic diagram, to further enable an AR see‐through viewing as well as a compact form factor, the system structure must be reconfigured.

The concept of the proposed AR near‐eye display is shown in Figure 1D. To achieve an optical see‐through viewing for AR applications, an off‐axis projection‐type display scheme is adopted in this work, and an optical combiner (OC) is deliberately introduced into the system structure, merging the display light rays from image source and the see‐through light rays from outside real scenes simultaneously. The display rays are projected off‐axis onto the OC surface, and then focused into a viewpoint at the center of eye pupil domain. The see‐through rays pass directly through the OC without being modulated and then entering the human eye. The other components, excepting the OC, are all positioned off‐axis to avoid obstructing the see‐through lights. It is noted that the very dense viewpoints are distributed in two dimensions on the eye pupil plane, while Figure 1D depicts only three bundles of beams corresponding to specific and discrete viewpoints to facilitate illustration.

A reflective‐type SLM based on liquid‐crystal‐on‐silicon (LCoS) technology is utilized as the image source here. Compared to transmissive SLMs, LCoS‐SLMs have higher resolution, contrast, frame rate and light efficiency, as well as a more compact form factor.^[^ ^29^, ^30^ ^]^ The LCoS panel modulates the orientation of liquid crystal (LC) molecules pixel‐by‐pixel to alter the polarization state of the illumination beam, thereby encoding image information,^[^ ^31^ ^]^ with the LCoS adopting a vertical alignment (VA) LC mode to achieve high contrast.^[^ ^32^ ^]^ Additionally, a polarizing beamsplitter (PBS) is required to split the incident (illumination) and reflected (modulation) beams of LCoS panel. In this work, a PBS based on wire grid polarizer is chosen to accommodate a large viewing eyebox and a wide FOV.

To demonstrate the proposed display more intuitively, the side view of the system structure is shown in Figure 1E,F, where the illumination unit and the display unit of LCoS‐SLM are depicted separately to clearly illustrate the light paths. The directions of the display light paths are indicated by the red arrow lines. Since this projection‐type display results in a highly off‐axis nonsymmetric system structure, it induces a larger system form factor and much greater unconventional nonsymmetric aberrations. To minimize the volume and design complexity of the off‐axis part, the intermediate image points are deliberately created between the illumination points and the viewpoints, effectively reducing the spatial volume occupied by the light beams. Several mirrors (M1, M2 and M3) are used to fold the light paths, ensuring a compact and desired system structure. The aperture stop of this display is positioned at the incidence plane of LCoS, fully utilizing the active area of the SLM panel for each viewpoint, and eliminating the stray lights. In addition, regarding the illumination beams of LCoS‐SLM, to achieve a uniform modulation, and avoid the pixels crosstalk, a collimation lens L2 is placed between the illumination source and the LCoS panel. Correspondingly, after the reflections by LCoS, a collimation lens L1 is also required between the LCoS panel and the intermediate image points.

### 2.1 Freeform Optics

Although a large DOF, large eyebox, and wide FOV may be possibly realized using the above proposed system working scheme, it is very challenging to achieve a high display performance as well as a compact form factor. In addition, as the system is highly nonsymmetric, significant aberrations with unconventional field‐dependence properties appear,^[^ ^33^, ^34^ ^]^ which are difficult to be corrected using traditional spherical and aspherical surfaces, thus greatly affecting the actual display performance, and limiting the actual eyebox or FOV.

To solve this problem, freeform optical elements are used in the design. Compared to conventional rotational symmetric surfaces, freeform surfaces are described using more parameters, which have much higher degree of design freedom, and much stronger abilities to correct aberrations, especially for the off‐axis nonsymmetric aberrations, achieving excellent system performance for systems with both a large eyebox and wide FOV.^[^ ^35^, ^36^ ^]^ Meanwhile, freeform optics can dramatically decrease the number of elements required in the optical system, and reduce the system volume and weights, achieving compact form factor.^[^ ^37^, ^38^ ^]^

**Figure 2:**
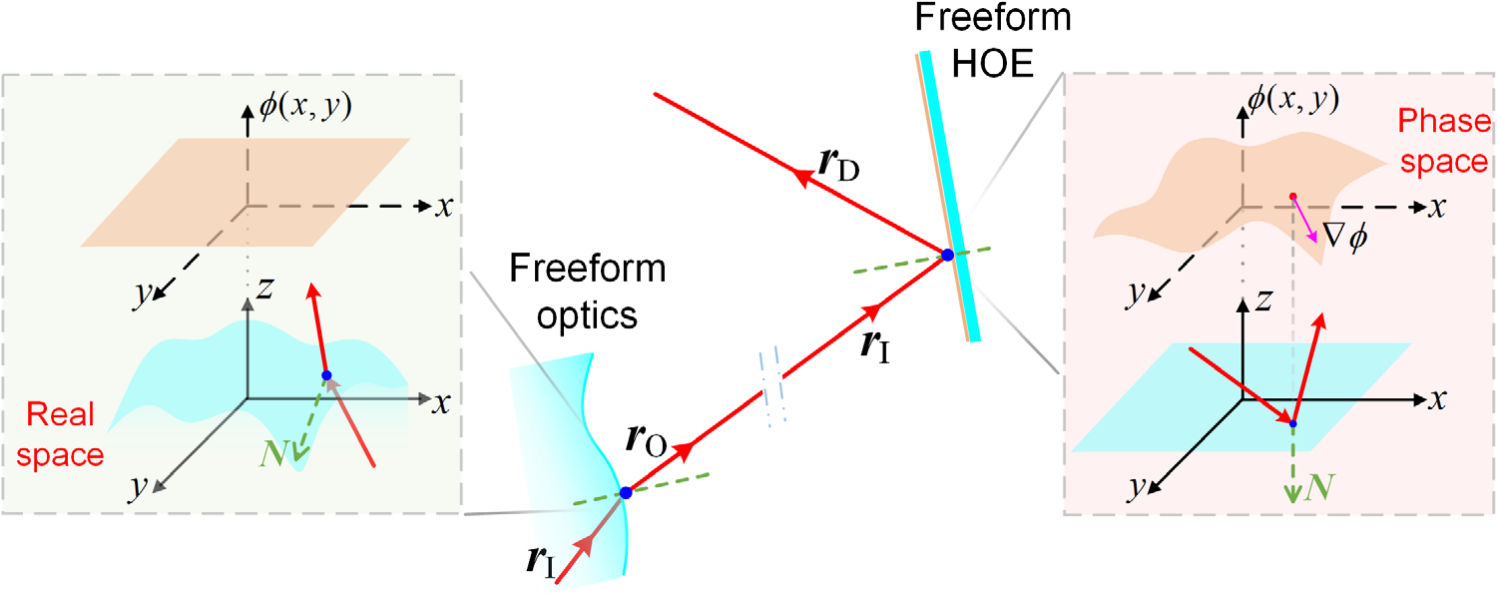
Schematic diagram of cooperatively designing freeform optics and freeform holographic optics.

Freeform surfaces are generally described by a sag function *z*(*x*, *y*) that represents the surface height at the substrate coordinate (*x*, *y*) in real space relative to the surface vertex, as shown in **Figure** **2**. The analytical mathematical expressions can describe the *z*(*x*, *y*), which mostly has non‐rotational symmetric terms (such as the polynomials or basis functions) overlay onto the spherical or conic base surface, such as XY polynomials, Zernike polynomials, orthogonal polynomials, NURBS, etc. In this work, the XY polynomials freeform surface up to the 8th order (Equation (1)) is employed to describe *z*(*x*, *y*) with a base conic, and the odd‐order terms of *x* are not used. Compared to Zernike polynomial or NURBS, the XY polynomial offers a simpler mathematical description. Being defined in Cartesian coordinates, the XY polynomial is well‐suited for modeling the freeform surface with a rectangular aperture, whereas the Zernike polynomial is more suitable to describe the surface shape defined over a circular aperture, in order to use the orthogonal property. The use of NURBS surface may decrease the ray tracing efficiency during the design and analysis. Moreover, the XY polynomial surface description is more compatible with fabrication techniques such as single point diamond turning (SPDT) or computer numerical control (CNC) machining.

(1) $$\begin{array}{cc} z\left(x,y\right) & =\frac{c\left(x^{2}+y^{2}\right)}{1+\sqrt{1-\left(1+k\right)c^{2}\left(x^{2}+y^{2}\right)}}\\ & \;+\sum \sum C_{i,j}x^{i}y^{j},\;2\leq i+j\leq 8 \end{array}$$

where *k* is the conic constant, and *c* is the curvature. (*x*, *y*) is the local coordinates of the substrate surface, and *C_i_*_,_ *_j_* is the coefficients of high‐order terms. In this way, the normal vector ***N*** of freeform surface can be easily calculated.

### 2.2 Freeform Holographic Optics

The optical functions achieved by the OC involve simultaneously merging the display light rays from the image source and the see‐through rays from the real‐world scenes. When selecting an OC, it should possess high transmittance and induce no distortions for the see‐through rays, have high modulation ability for the display rays, and feature a compact and esthetic form factor.^[^ ^39^ ^]^ Various types of optical elements, such as freeform half‐mirror,^[^ ^40^ ^]^ diffractive optical element (DOE),^[^ ^41^ ^]^ metasurface,^[^ ^42^ ^]^ HOE,^[^ ^43^ ^]^ etc., can be employed as the OC.

For the freeform half‐mirror, to implement a desired off‐axis system architecture, the freeform surface must be tilted at certain angle (typically several tens of degrees) with respect to the viewing axis of human eye;^[^ ^44^ ^]^ however, such a tilted‐combiner would increase the system volume and compromise the form factor. Additionally, to guarantee good viewing performance and reduce distortion in the see‐through path, the other side of the freeform OC element should also be a freeform surface after special optimization, which would increase the manufacture cost and difficulty. In contrast, the diffractive element can achieve the off‐axis structure simply by utilizing a linear phase term in the *y* direction, without tilting surface substrate, thereby ensuring system compactness and improved aesthetics.

Because the fabrication cost of DOE or metasurface is generally higher than that of HOE, in this work, comprehensively considering cost, functionality, and feasibility, a reflective HOE is preferably selected as the OC because of its excellent and unique characteristics, such as waves modulation, selectivity, thin and lightweight form factor, easy to fabrication, as well as low cost.^[^ ^45^, ^46^ ^]^ Owing to the selectivity and flat substrate of HOE, the see‐through light rays from real‐world scenes can also pass through the HOE without introducing aberrations and distortions or attenuating brightness, thereby realizing better optical see‐through viewing than other types of OC. In addition, compared to a transmissive HOE, the reflective HOE enables a more practical and compact system architecture for implementing AR see‐through near‐eye displays.

Holographic element is fabricated by the interference of mutually coherent light waves inside the photosensitive medium (e.g., photopolymer, etc.),^[^ ^47^, ^48^, ^49^ ^]^ which is intrinsically different from computer‐generated hologram (CGH) fabricated by lithographic process for the surface or wavefront metrology applications.^[^ ^50^ ^]^ Traditional HOEs are mostly fabricated using the spherical (planar) waves, where the degree of design freeform only includes the point source coordinates of waves, imposing strong restrictions on the period distribution of the holographic grating. For this reason, conventional HOEs have very limited abilities to modulate waves, while can only achieve a good mapping between only one pair of specific object‐image points (i.e., the recording points of HOE), and other points would have very severe aberrations and low diffraction efficiency.

To breakthrough these limitations, freeform wave can be intentionally adopted as the recording waves of HOE, which can be referred to freeform HOE (FHOE) here. Compared to spherical waves, the wavefront profile of freeform waves becomes more complex, which gives a higher degree of design freedom. This could formulate a more complex distribution for the holographic grating period (or frequency) on the substrate surface, offering stronger abilities to modulate waves and correct aberrations. In the experiment setups, to generate the desired freeform recording wave for FHOE, the wavefront correction element, such as freeform optics, phase‐only SLM, etc., needs to be incorporated into the holographic recording system. Through the optimized combination of the correction element and the simple spherical waves, the recording waves required by FHOE can be obtained.

An analytical phase function *ϕ*_G_(*x*, *y*) can also be expressed by the linear combination of polynomials or radial basis function, such as XY polynomials, Zernike polynomials, NURBS, etc. In this research, similar to designing freeform optics, a phase function *ϕ*_G_(*x*, *y*) described by XY polynomials up to the 10th order is used to characterize FHOE, and the odd‐order terms of *x* are also not used, that is

(2) $$\phi_{G}\left(x,y\right)=\frac{2\pi }{\lambda_{c}}\sum \sum A_{i,j}x^{i}y^{j},\;1\leq i+j\leq 10$$

where *λ*_c_ is the recording wavelength of the holographic element, and *A_i_*_,_ *_j_* is the coefficients of phase terms. (*x*, *y*) are the local coordinates of the substrate surface, where the coordinates (*x*, *y*) in real space map to that of the phase space one‐by‐one, as shown in Figure 2. The phase gradient ∇*ϕ*(*x*, *y*) represents the spatial frequency (reciprocal of period) distribution of the holographic grating along the *x* and *y* directions on the substrate surface. For traditional HOEs fabricated by the spherical waves, the coefficients *A_i_*_,_ *_j_* are coupled each other, which greatly limits the degree of design freedom. In contrast, the design of FHOE enables each term *A_i_*_,_ *_j_* to be independent, thus offering stronger capabilities to modulate waves.

Although traditional HOE features common properties with FHOE, such as multiplexability, selectivity, lightweight form factor, and see‐through viewing, etc., in terms of design freedom and aberration correction capability, FHOE fabricated using freeform waves is significantly superior to traditional HOE fabricated using spherical waves. To achieve the system specifications (such as FOV and viewing eyebox) comparable to that enabled by FHOE, traditional HOE tends to increase design difficulty and compromise the system form factor as well as number of elements to some extent. In other words, for the same system form factor and number of elements, the OC using traditional HOE would compromise the FOV, viewing eyebox, and DOF. In addition, freeform half‐mirror can provide a degree of design freedom and aberration correction capability comparable to that of FHOE in theory, and when used as the OC, it can achieve display performance consistent with that enabled by FHOE. However, the manufacture cost of using the FHOE as OC is lower than that of a freeform OC element.

Based on the above discussions, instead of traditional HOE or surface, as well as other diffractive elements, FHOE is well suited as the OC for the proposed display system, achieving both high display performance as well as compact system form factor.

### 2.3 Co‐Design of Freeform Optics and Freeform Holographic Optics

Since the proposed display scheme simultaneously comprises the geometric (freeform) optics and the diffractive (holographic) optics, cooperatively modeling and simulating these optics in a unified framework is essential to effectively design and optimize this system. Additionally, since a large number of light rays need to be sampled for this design, which requires a highly efficient design process, enabling the rapid analysis and evaluations. Regarding the design of holographic optics, if the rigorous wave optics theory is used, the system modeling and design processes would be very time consuming and require extensive computing load.^[^ ^51^, ^52^, ^53^ ^]^

To solve the above problem, in this research, we use an approach based on ray tracing to implement the whole system design, as shown in Figure 2. Assuming that the thickness of holographic medium layer is very thin, the ray tracing can effectively simulate the light wave modulation of HOE with a phase function *ϕ*_G_(*x*, *y*), which can be derived from the generalized Snell's law.

(3) $$n_{I}\left(N\times r_{I}\right)=n_{D}\left(N\times r_{D}\right)+\frac{m\lambda }{2\pi }\left(N\times \nabla \phi_{G}\left(x,y\right)\right)$$

where *n*_I_ and *n*_D_ are the refractive index of the incident and diffracted sides, respectively. *λ* the wavelength of the incident rays. *m* indicates the diffraction order, and it is set to be +1 for the holographic element. In this work, the substrate of the holographic element is planar, and thus, ***N***, the normal to the substrate surface, is a constant vector. *ϕ*_G_(*x*, *y*) can be described using the analytical mathematic form, such as polynomials or basis functions. The schematic diagram of ray tracing for the holographic optics is illustrated in Figure 2, where the substate surface is in real space, and the phase function is in a virtual 3D space, referred to as the phase space.

By setting the phase function as a constant, Equation (3) can implement the ray tracing for the geometric optical elements such as freeform optics. In contrast to holographic elements, freeform optics modulate ray directions through continuous changes in the substrate surface shape, which requires computing the gradient of sag function *z*(*x*, *y*) to acquire the normal vector ***N*** during the tracing. The schematic diagram of ray tracing for the freeform optics is illustrated in Figure 2. As described above, the integrated design of freeform optics and freeform holographic optics can be implemented through ray tracing within a unified computing framework, achieving the efficient design for the proposed display. In addition, since this design requires a high speed and accuracy for the ray tracing, the analytical mathematic form is used to describe both the phase function *ϕ*_G_(*x*, *y*) and the sag function *z*(*x*, *y*), which can rapidly and precisely compute their partial derivative. The optimization for both freeform and holographic optics can be performed employing the multi‐parameter approach to find an optimal solution space, reaching the desirable performance and boundary constraints.

It is noted that the ray tracing for the freeform holographic optics can also be implemented using a grating vector field ***Ψ***_G_(*x*, *y*) that is derived from the recording wavevectors. Meanwhile, the optimization process can also be performed using the multi‐parameter approach. However, when employing the vector field ***Ψ***_G_ to characterize FHOE, the holographic recording system of FHOE can only be designed through an iterative optimization approach. This means that the holographic display/imaging systems based on FHOE and the holographic recording system of FHOE cannot be jointly designed and optimized to further regulate the diffraction efficiency of the holographic grating. Detailed interpretations about this issue are described in the Section 3 (Supporting Information).

### 2.4 Holographic Recording System for Freeform HOE

The FHOE with phase function *ϕ*_G_(*x*, *y*) can be completely fabricated using the spatially continuous recording waves. Regarding the degree of design freedom, freeform optics described by the XY polynomials in Equation (1) is comparable to the FHOE described by the phase function in Equation (2). In this work, the freeform mirror is selected to generate the freeform recording wave required by FHOE in the holographic recording system. Compared to the phase‐only SLMs or freeform lens, freeform mirror can achieve the spatially continuous modulations for the wavefront profile, effectively suppress stray lights, and reduce system complexity and recording time duration.

In order to generate the recording waves (the signal wave and the reference wave, respectively) that can reconstruct the required phase function *ϕ*_G_(*x*, *y*), the holographic recording system of FHOE needs to be additionally and specifically designed. The design target is to find the locations of the two light sources and freeform mirror, as well as the freeform surface shape, However, it is very difficult to design the corresponding system based on the principle of wavefront interference. Here we convert this design problem into an imaging system design task based on the principle of reversibility of light and direct ray tracing. The optical layout of the recording system is equivalent to an imaging system. The spherical wave coming from one point source (object point) is modulated by the FHOE (with phase function *ϕ*_G_(*x*, *y*)) and freeform mirror, and is finally redirected to the other point source (image point), and realize stigmatic imaging. This imaging system (recording system) can be efficiently designed using multi‐parameter optimization approach with target maximizing the imaging performance.

### 2.5 Joint Optimization

Using the above proposed method, the holographic recording system of FHOE can be obtained. However, when employing common performance metrics (e.g., spot size) and system constraints (e.g., avoiding structural interference and stray lights), the recording system design may have multiple solutions, each corresponding to a different system structure. Although these solutions can all generate the required FHOE, the distributions (magnitude and uniformity) of diffraction efficiency for the FHOE when applied to specific imaging or display systems are different. To obtain the optimal recording structure, the regulation of diffraction efficiency for the FHOE should also be involved into the loss functions during the system design.

**Figure 3:**
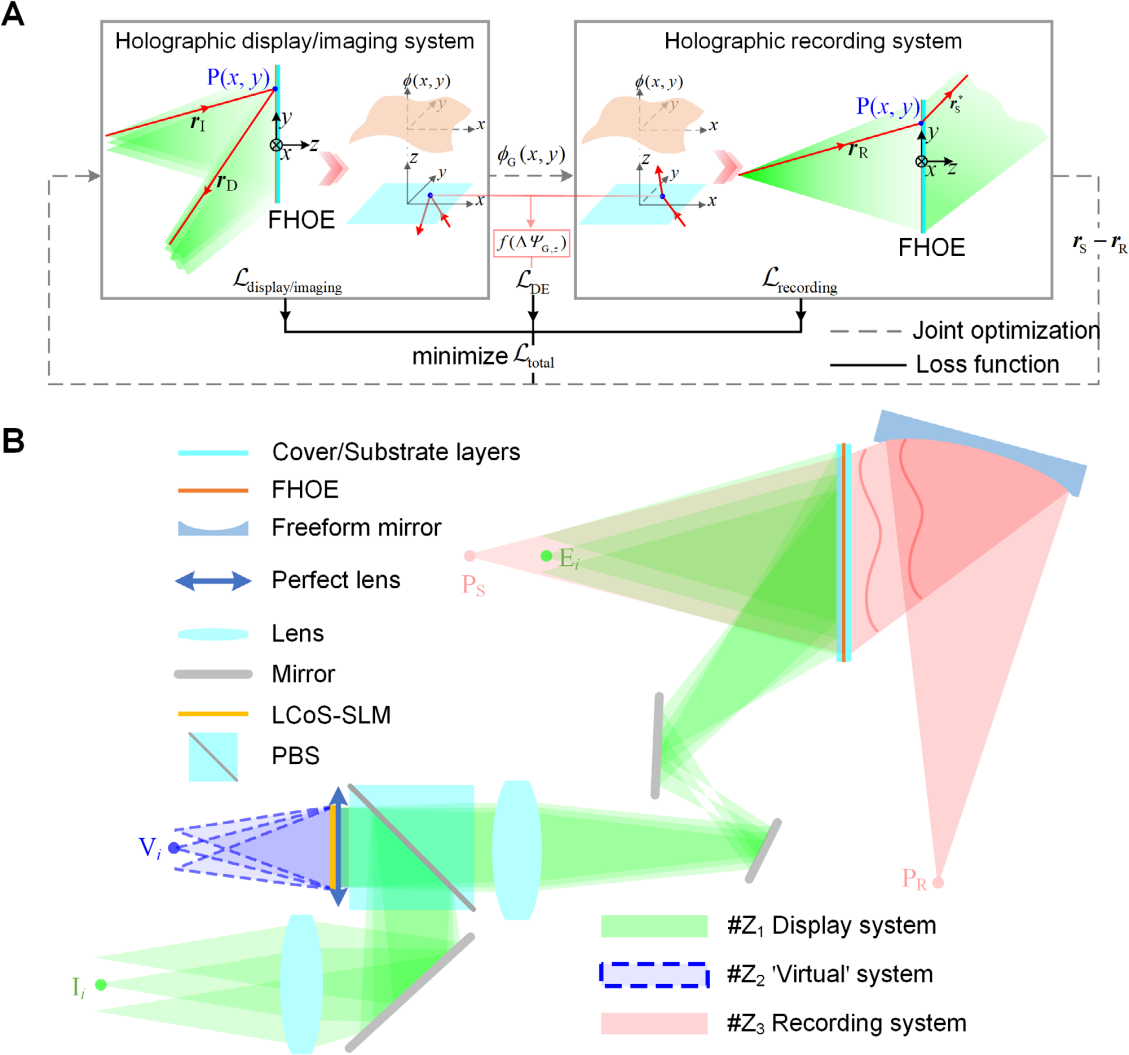
The entire design framework. A) Joint optimization. B) Designs of the display system and the holographic recording system using multi‐configurations. Display system in configuration Z_1_. ‘Virtual’ system in configuration Z_2_. Holographic recording system for FHOE in configuration Z_3_.

As shown in **Figure** **3**A, for the imaging or display optical system based on FHOE, when a light ray ***r***_I_ intersects at the FHOE substrate surface at a point P(*x*, *y*), the diffraction efficiency of the 1st order diffracted ray ***r***_D_ is determined by the grating vector ***Ψ***_G_(*x*, *y*) at this point P(*x*, *y*), that can be derived from the recording waves ***r***_S_(*x*, *y*) and ***r***_R_(*x*, *y*), that is

(4) $$\Psi_{G}\left(x,y\right)=r_{S}\left(x,y\right)-r_{R}\left(x,y\right)$$

The extent of attenuation in diffraction efficiency can be characterized by the Equation (5).

(5) $$\Delta \Psi_{G,z}\left(x,y\right)=\left|r_{D,z}\left(x,y\right)-\Psi_{G,z}\left(x,y\right)-r_{I,z}\left(x,y\right)\right|$$

Since a phase function *ϕ*_G_(*x*, *y*) is used to characterize the FHOE in this work, the phase gradient ∇*ϕ*(*x*, *y*) only represents the spatial frequency distribution of the holographic grating along the *x* and *y* directions on the substrate surface. The component *Ψ*_G,_ *_z_* (*x*, *y*), which represents the spatial frequency along the *z* axis, cannot be obtained from the phase function *ϕ*_G_(*x*, *y*), meaning that directly employing *ϕ*_G_(*x*, *y*) does not achieve the regulation of diffraction efficiency for the FHOE. However, the parameter *Ψ*_G,_ *_z_* (*x*, *y*) at the point P(*x*, *y*) can be exactly computed by directly tracing the light rays in the holographic recording system of FHOE. Referring to the Equation (4), the *Ψ*_G,_ *_z_* (*x*, *y*) can be expressed as

(6) $$\Psi_{G,z}\left(x,y\right)=r_{S,z}\left(x,y\right)-r_{R,z}\left(x,y\right)$$

where the parameters *r*_S,_ *_z_* (*x*, *y*) and *r*_R,_ *_z_* (*x*, *y*) can be easily acquired by tracing the recording light rays $r_{S}^{*}$ and $r_{R}$ that intersect at the point P(*x*, *y*) on the FHOE substrate surface, as shown in Figure 3A. When the parameter Δ*Ψ*_G,_ *_z_* (*x*, *y*) equals zero, fully satisfying the Bragg condition, the diffraction efficiency of the diffracted light ray ***r***_D_ reaches the highest. However, deviations in the wavelength or angle of the incident wave from the recording condition of the holographic grating would result in a non‐zero value for Δ*Ψ*_G,_ *_z_*, leading to the attenuation of diffraction efficiency.^[^ ^51^, ^53^ ^]^

The data in Equation (5) are all associated with both the holographic display system and the holographic recording system, and can be exactly obtained through direct ray tracing. Consequently, employing the parameter Δ*Ψ*_G,_ *_z_* to control the diffraction efficiency of the light rays diffracted by FHOE requires the joint optimization for these two types of systems. As illustrated in Figure 3A, the distribution (magnitude and uniformity) of diffraction efficiency can be numerically regulated during the joint optimization by constructing a loss functions ℒ_DE_, which is a function of ∆*Ψ*_G,_ *_z_*. Regarding the entire design framework shown in Figure 3A, the design of holographic recording system is implemented according to the optimized phase function *ϕ*_G_(*x*, *y*) of FHOE tailored for the specific holographic display system, and the recording waves ***r***_S_ and ***r***_R_ acquired from the holographic recording system can enable the effective fabrication for the required FHOE. The joint optimization is intentionally conducted to achieve the desired display performance, diffraction efficiency, system constraints as well as fabrication requirements.

In this work, the proposed design framework is implemented using multi‐configurations, as shown in Figure 3B. Configuration Z_1_ is designed to obtain the desired display system by modeling the mapping between the viewpoints E*_i_* and the illumination points I*_i_*. Regarding any of viewpoints E*_i_*, the quasi‐collimation illumination waves are required for the LCoS panel, ensuring a uniform and accurate modulations by LCoS panel. This can be achieved by placing a perfect lens at the LCoS plane during the system design. The spot size formed by the perfect lens when focusing the incident waves onto the LCoS can represent the collimation degree of the illumination waves; a smaller spot size corresponds to higher collimation. In this work, the perfect lens is used solely as a theoretical concept in optics, capable of realizing ideal point‐by‐point imaging without aberrations, to evaluate the collimation of illumination waves for the LCoS panel, which does not have practical physics. Configuration Z_2_ is designed to obtain the quasi‐planar illumination waves incident on the LCoS panel by modeling the mapping between the viewpoints E*_i_* and the “virtual” image points V*_i_*. During the optimization, the weight value of loss function corresponding to the configuration Z_2_ is set significantly smaller than that of the configuration Z_1_. In addition, several boundary constraints must be considered to ensure a feasible system structure and desired display requirements, such as constraining the incidence angle tolerance (less than 12°) of the illumination waves on the LCoS plane,^[^ ^54^ ^]^ and simulating the emission characteristics (telecentric illumination) of the illumination source, etc.

The holographic recording system of FHOE is designed in another configuration Z_3_. The design target is to obtain the recording waves required by FHOE through the optimized combination of the simple spherical waves and the freeform mirror. In this research, FHOE is fabricated based on the conjugate recording principle. For the display system, the FHOE actually generates a set of spherical waves converging at the eyebox plane. To ensure a high diffraction efficiency for these display beams, and facilitate the joint optimization to efficiently find the optimal solution for regulating the diffraction efficiency, the recording wave of the FHOE should encompass these spherical waves and match their wavefront profile as closely as possible. As shown in Figure 3B, a divergent spherical wave emitted from a point source P_R_ near the eyebox plane of the display system, is selected as the one recording wave of the FHOE. This spherical wave is modulated by FHOE with a phase function *ϕ*_G_(*x*, *y*) into a freeform wave. Then, a freeform mirror modulates this freeform wave into a spherical wave converging at a point source P_S_. The above design process can be enabled by optimizing the freeform surface shape and the position coordinates of each component. Meanwhile, some system constraints must be constructed to avoid stray lights, lights obscuration and structure interference in this recording system, ensuring the effective fabrication for the holographic grating. During the experimental fabrication of FHOE, the spherical waves emitted from these two points sources {P_R_, P_S_}, after modulated by the freeform mirror, can reconstruct the required recording waves on the FHOE surface.

Tracing the feature light rays in configurations Z_1_ and Z_3_ can construct the loss function ℒ_DE_ related to the diffraction efficiency, aiming to regulate the diffraction efficiency of FHOE functioning on the display rays for the different viewpoints E*_i_* and pupil aperture coordinates, thus achieving a high and uniform display brightness across the viewing eyebox and FOV range. Regarding the sampled viewpoints E*_i_*, the feature rays are sampled densely within the range of pupil aperture. The root‐mean‐square (RMS) and standard deviation (SD) of the parameters ∆*Ψ*_G,_ *_z_* (*x*, *y*) corresponding to the sampled feature rays are used to construct the loss function ℒ_DE_. Then, the display system in configuration Z_1_ and Z_2_, as well as the holographic recording system in configuration Z_3_ conduct the joint optimization to obtain the optimal system solution for both by minimizing the total loss functions that consider system performance, diffraction efficiency, boundary constraints, and fabrication requirements. Detailed descriptions of the loss function ℒ_DE_ for regulating diffraction efficiency and the boundary constraints for configurations Z_1_ and Z_3_ are provided in Section S4 (Supporting Information). A comprehensive analysis and evaluation of the diffraction efficiency of FHOE is presented in Section S8 (Supporting Information).

## 3 Display Prototype and Results

**Figure 4:**
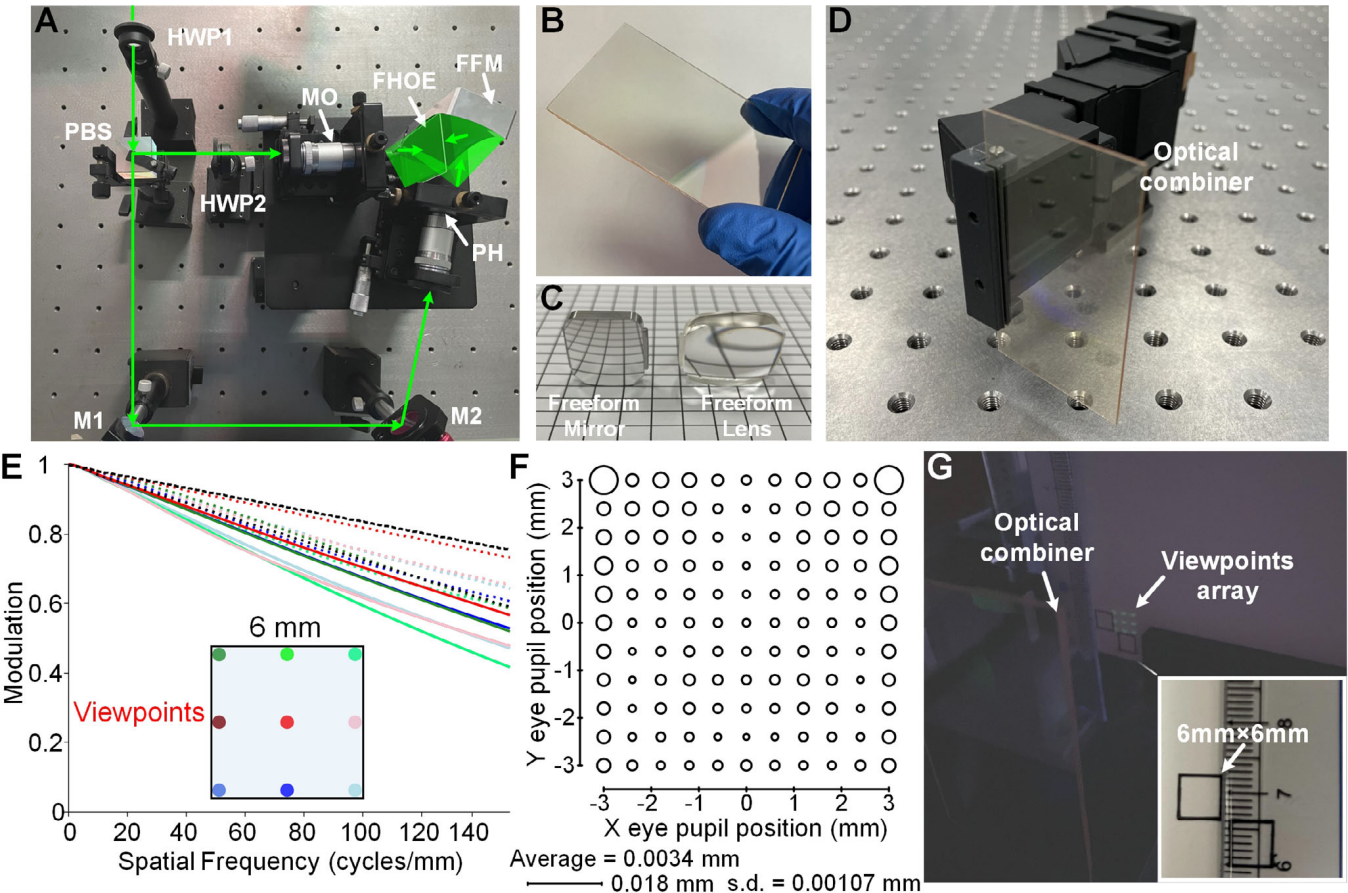
Display prototype. A) Holographic recording system setup for FHOE. B) Fabricated FHOE. C) Freeform lens and mirror. D) Display system prototype. E) MTF curves for the nine typical sampled viewpoints. F) RMS spot size of the densely sampled viewpoints array. G) Formed viewpoints array captured by the camera lens.

For the holographic recording system design of FHOE, the experimental recording setup is shown in **Figure** **4**A, where freeform mirror (FFM) modulates the recording wave. The fabricated FHOE serving as the OC is shown in Figure 4B, with the freeform lens and mirror shown in Figure 4C, and the display system prototype illustrated in Figure 4D. The LCoS has a resolution of 1920×1080 pixels, a size of 0.69 inches, a pixel size of 8 µm, and a frame rate of 360 Hz, and is supplied by Nanjing SmartVision Electronics Co., Ltd. The illumination source is a micro‐OLED with a monochromatic green display, a luminance of up to 20000 nits, a pixel size of 7.2 µm, a resolution of 2560×2560 pixels, a contrast ratio of 500000:1, and a frame rate of 90 Hz, supplied by SeeYA Technology.

In general, the micro‐OLED panel presents a large viewing angle (greater than 70 degrees), which means that it emits lights over a wide angular range that exceeds the effective emission angle required by the display system. The portion of illumination light that exceeds this effective emission angle is categorized as stray light, which can be validly eliminated using a custom‐designed mechanical structure. Since the aperture stop is positioned at the LCoS plane during the display system design, the active area of the LCoS panel fully determines the effective emission angle of the micro‐OLED panel and restricts the valid bundle width of illumination beams. The illumination light outside the effective emission angle does not reach the active area of the LCoS panel. Therefore, during the system assembly, a custom mechanical structure is utilized to mount LCoS panel, in which the structural region corresponding to the active area of the LCoS panel is designed as an opening aperture. The structural surface is treated through anodizing and blackening processes to significantly enhance light absorption and minimum light reflection. When the micro‐OLED panel illuminates the LCoS panel, stray light outside the effective emission angle would be absorbed by the blackened structure, thereby having less impact on the display performance.

The measured diffraction efficiency of the HOE is 80.6% for the chief ray of the central viewpoint. In practice, the diffraction efficiency of the fabricated volume holographic grating is typically below 100%, not only due to Bragg mismatch introduced during the system design, but also largely influenced by various fabrication‐related factors, such as the intensity of the recording beams, recording dosage, post‐processing conditions, absorption in the recording medium, and fabrication and assembly errors. For optical systems involving diffractive optical elements, it is important to consider the impact of zero‐order light during the system design, as it may introduce severe stray light and degrade display performance. In general, the zero‐order light originates from the direct refraction or reflection of incident light on the surface of the diffractive element based on the traditional Snell's law or the law of reflection. In the proposed display system employing a reflective HOE as the OC, in the case of non‐100% diffraction efficiency, the incident light on the HOE surface generates not only the desired diffracted light, but primarily the zero‐order reflection light, along with residual energy distributed among other diffracted orders.

In this work, to ensure that the zero‐order light deviates from the intended viewing eyebox region, an off‐axis system architecture is adopted, and an upright‐oriented reflective HOE is employed as the OC. When the display beam is obliquely projected onto the HOE surface from an off‐axis position, the desired diffracted light will be redirected toward the intended viewing eyebox plane, which is co‐axis and parallel to the HOE surface. The zero‐order light, being directly reflected by the HOE surface or travelling through the OC, will deviate significantly from the viewing eyebox region and will not enter the eyebox. Meanwhile, other diffracted orders will also deviate from the desired normal light path, and has minimal impact on the display performance due to very low energy. Therefore, in the proposed display system, the adoption of an off‐axis system structure can effectively eliminate the impact of zero‐order and unwanted diffracted order light on normal display performance.

Using the proposed system scheme, each self‐emissive pixel on the micro‐OLED panel is an illumination point, corresponding to a viewpoint. In this way, a highly dense viewpoint array can be realized and each viewpoint can be controlled individually. The modulation transfer function (MTF) curves for the nine typical sampled viewpoints are shown in Figure 4E. The RMS spot size of densely sampled viewpoints array is shown in Figure 4F. The viewpoints array captured by the camera across the 6 mm×6 mm range is shown in Figure 4G, merely demonstrating the sampled nine viewpoints here. Regarding the average eye pupil size of 4 mm, the viewing eyebox can be expanded to 10 mm×10 mm. It should be noted that the viewpoint sizes have been deliberately enlarged to facilitate direct capturing by the camera lens. The dynamic switching of densely sampled viewpoints is demonstrated in Video S1 (Supporting Information).

**Figure 5:**
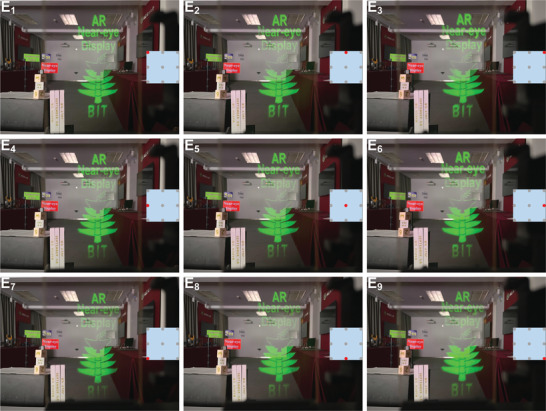
Captured virtual images at the different viewpoint positions.

At the different viewpoint positions E*_i_* (1≤*i*≤9), the captured virtual images are shown in **Figure** **5** respectively. The diagonal viewing FOV for each viewpoint is larger than 50°. The Video S2 (Supporting Information) shows the movement process of the camera, where the viewpoint dynamically follows the camera.

**Figure 6:**
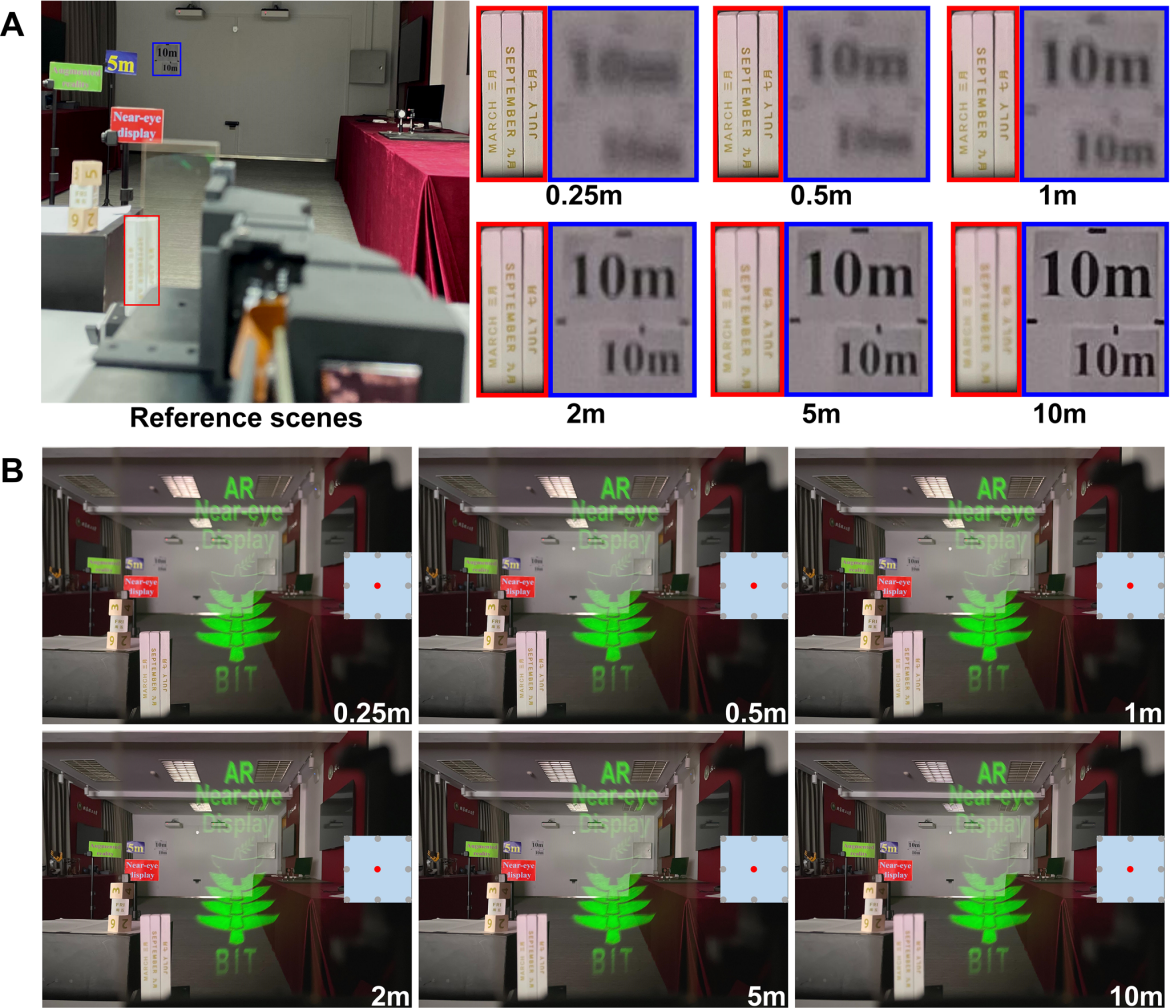
Change in camera focus. A) Reference scene. B) Captured virtual images at the different depths for the viewpoint position E_5_.

The reference scenes are shown in **Figure** **6**A. As the camera changes the focus, the targets located at a depth of 0.25 m (marked by the red line box) and 10 m (marked by the blue line box) are enlarged and shown in Figure 6A, respectively. For the viewpoint E_5_ located at the center of eyebox, when the camera changes the focus from 0.25 to 10 m, the captured virtual images are shown in Figure 6B, which keeps good consistence in the image quality across different depths. In this way, large DOF is realized for the near‐eye display system. The Video S3 (Supporting Information, the video has been compressed) demonstrates this process of changing the camera's focus. In addition, at the other viewpoint position E*_i_*, the captured virtual images at the different depths are demonstrated in Figures S9–S17 (Supporting Information)

## 4 Conclusion

In summary, we have proposed an AR near‐eye display that achieves a large DOF, viewing eyebox, and wide FOV simultaneously. The DOF range is extended by directly reducing the effective exit‐pupil into a viewpoint at the center of eye pupil, and the eyebox is expanded by dynamically and continuously shifting the viewpoints in space to accommodate the movement of eye pupil. The overall system form factor is relatively compact and does not require the complex mechanical steering or moving devices. The display results, featuring a DOF from 0.25 to 10 m, a 50° large FOV and a 10 mm×10 mm viewing eyebox, as well as good optical see‐through viewing, are demonstrated in detail, verifying the effectiveness of the proposed scheme. To our knowledge, regarding the AR near‐eye displays realizing a large DOF, the proposed display scheme in this work can achieve, for the first time, a dynamic and continuous expansion of the viewing eyebox without using any deflection or moving devices. The proposed system scheme and design provides a breakthrough for AR near‐eye display, and can be used in various areas such as industry, defense, education, medicine, entertainment, etc.

Additionally, a pupil‐tracking module can be further integrated into this display to detect the positions of eye pupil in real time, and operates with the LCoS and OLED panels synchronously, enabling the display viewpoints to dynamically track the eye pupil and realize gaze‐match. The signal capture of pupil positions, as well as the triggering and synchronization of electronic signals required by several devices in the display prototype are outside the research scope of this work. Similar to the HOE, the liquid crystal polarization hologram (LCPH) is also fabricated using coherent laser beams, provides optical functionalities such as converging or diverging light waves, and demonstrates good optical see‐through viewing. Relevant research on using LCPH for AR near‐eye displays has been conducted in academia, and the design framework proposed in this work is also applicable to the LCPH after appropriate adjustments. Therefore, LCPH can serve as a candidate OC for the proposed display system.

The currently built prototype is designed only for a monochromatic green display, and a full‐color display can also be implemented by using a full‐color FHOEs and a color illumination source, which illuminates the LCoS in a field sequential color (FSC) mode with high frequency.^[^ ^32^ ^]^ The color illumination source can be available commercially, such as full RGB OLED panel. Based on the wavelength multiplexability of the holographic grating, the full‐color HOEs can be fabricated by either laminating three layers of substrate for monochromatic HOEs (red, green, and blue) or multiplexing three color HOEs into a single layer of color‐photopolymer. Owing to the wavelength selectivity of the holographic grating, each monochromatic HOE functions only on the display beam of its corresponding color channel. In the design of full‐color HOEs, according to the generalized Snell's law, and under the assumption that the chromatic dispersion of the medium is ignored, HOE acts equivalently as an ideal diffractive phase surface. In this case, for each monochromatic HOE, when the reconstruction wavelength is identical to the recording wavelength, or the ratio of recording and reconstruction wavelengths are all equal, the phase function only needs to be designed for one color channel, and it can also accommodate the other two without additional designs. However, the above situation does not hold in practice, and there is a different wavelength ratio between the recording and reconstruction stages for each monochromatic HOE. Therefore, the phase functions of three‐color channels must be designed independently, that is, the holographic display and recording systems for each color channel are designed in different configurations Z*_i_*. The joint optimization is then conducted on all these configurations to control the chromatic aberration and regulate the diffraction efficiency among the three‐color channels. The design process is identical to that of the monochromatic green display described in this work, with additional chromatic aberration correction required for the optimized optics, such as chromatic lenses.

## 5 Experimental Section

The holographic recording setup of FHOE is shown in Figure 4A, where the optical/mechanical components are mounted at the same base plate for the precise calibrations. The photopolymer, featuring high diffraction efficiency and stability, is employed as the recording medium of holographic element.^[^ ^55^ ^]^ It is provided by Beijing Spectrum Treasure Printing Technology. The 532 nm laser beam is used to fabricate the holographic grating. PBS splits the incident laser beam into the two recording beams. Half‐wave plate (HWP1) alters the intensity ratio of the two recording beams on the FHOE surface, and HWP2 ensures that the two recording beams have consistent polarization state. Freeform mirror (FFM) modulates the recording wave. The combination of the microscope objective (MO) and the pinhole (PH) generates a quasi‐spherical wave with high beam quality, where PH filters the diffraction noises and the stray lights. M1 and M2 fold the light paths.

Before fabricating the freeform holographic element, the photopolymer film layer, which serves as the holographic recording medium, is attached to a layer of substrate glass. In dark lab conditions, the holographic grating is fabricated by the two coherent recording beams inside the photopolymer based on the interference principle. After the exposing, post‐treatment processes are carried out to improve the stability and preservability of the holographic grating. The UV curing process for 5 mins is first conducted to consume the remaining monomers inside the photopolymer layer. Then, the baking process at 100 °C for 10 mins is conducted to improve the performance of grating. In practical applications, when the holographic grating is directly exposed to air, its microstructure may be damaged by dust, particles, water vapor, scratches, and other contaminants. To protect holographic grating, a cover glass is deliberately cemented to the photopolymer, which has already been attached to the substrate glass, after the exposure and post‐treatment processes. The cover glass functions equivalently to a flat glass plate, which would introduce a deflection of light rays. During system design, the cover and substrate glasses are precisely modeled based on their thickness and material properties, and ray deflections introduced by the glass plates are carefully taken into account. Both the cover and substrate glasses have a thickness of 0.7 mm, which can be further reduced depending on the capabilities of glass fabrication and cementation techniques. The glass material is K9.

The freeform optics are fabricated using the SPDT, with aluminum as the substrate material for the mirror and K22R as the lens material. In addition, to enhance the transmittance or reflectivity for the display beams, the optical surfaces are coated with anti‐reflection coatings on the lens surface and cover/substrate glass surface, and the high‐reflection coatings on the mirror surface.

## Conflict of Interest

The authors declare no conflict of interest.

## Author Contributions

Y.W. and T.Y. performed the system design. Y.W. conducted the experiment. Y.W. and T.Y. wrote the manuscript. Y.W., T.Y., X.L., D.C., and Y.W. shared their insights and contributed to discussions on the results. T.Y. and D.C. supervised the project.

## Supporting information



Supporting Information

Supplemental Video1

Supplemental Video2

Supplemental Video3

## Data Availability

The data that support the findings of this study are available from the corresponding author upon reasonable request.
