# Supplementary material for: Large Depth‐of‐Field, Large Eyebox, and Wide Field‐of‐View Freeform‐Holographic Augmented Reality Near‐Eye Display
Source: Adv Sci (Weinh). 2025 Jul 11;12(37):e08773. doi: 10.1002/advs.202508773 (PMC12499390; doi:10.1002/advs.202508773)
Supplement: Supplementary file 1 — Supporting Information [file ADVS-12-e08773-s003.pdf]

ADVANCED SCIENCE
Open Access

Supporting Information

for *Adv. Sci.*, DOI 10.1002/advs.202508773

Large Depth-of-Field, Large Eyebox, and Wide Field-of-View Freeform-Holographic Augmented Reality Near-Eye Display

*Yongdong Wang, Tong Yang**, *Xin Lyu, Dewen Cheng and Yongtian Wang*

Supporting Information:

Large depth-of-field, large eyebox, and wide field-of-view freeform-holographic augmented reality near-eye display

Yongdong Wang, Tong Yang, Xin Lyu, Dewen Cheng, Yongtian Wang*

Beijing Engineering Research Center of Mixed Reality and Advanced Display, School of Optics and Photonics, Beijing Institute of Technology, Beijing 100081, China
E-mail: yangtong@bit.edu.cn

S1 The limitations of the laser scanning projector (LSP) used as the image source

In some prior research on retinal projection display systems conducted in both academia and industry, laser scanning projector (LSP) has indeed been used as the image source. However, LSP generally imposes critical limitations on retinal projection displays with eyebox expansion, such as increased system volume, a limited number of viewpoints, etc.

A laser scanning projector is typically based on a dual-axis micro-electromechanical systems (MEMS) scanning mirror combined with three-color laser diodes. From a perspective of system design for retinal projection display, the point at which the laser beam intersects the MEMS scanning mirror can be considered an illumination point, which is conjugated to a viewpoint located at the center of eye pupil plane. In general, there are two approaches to expanding the viewing eyebox in retinal projection displays that use LSP:

1) One approach uses multiple LSPs, each creating a corresponding viewpoint on the eye pupil plane. However, since a single LSP occupies a certain volume, this method can generate only a limited number of viewpoints along a single dimension, resulting in a bulky system volume and high costs. Therefore, this approach is generally considered infeasible.
2) The other approach is to use a single LSP and employ special optical or mechanical devices to generate multiple viewpoints, such as a beam splitter (BS) array, Pancharatnam-Berry deflectors (PBD), fast steering mirror (FSM), moving device, and polarization controller, etc. However, these additional optical and mechanical devices still result in a complicated and bulky system form factor, and the number of viewpoints remains limited by the inherent characteristics of these devices.

Considering the above limitations, an LCoS panel is selected as the image source in this work, while a micro-OLED panel with high pixel density and luminance is selected as the illumination source, enabling the generation of ultra-dense viewpoints within a compact system structure.

S2 The polarizing beamsplitter (PBS)

The operation of the LCoS panel typically requires a polarizing beamsplitter (PBS). The LCoS panel modulates the orientation of the liquid crystal (LC) on a pixel-by-pixel basis to alter the polarization state of the incident beam, thereby encoding the image information. The PBS is used to separate the modulated and unmodulated beams of the LCoS panel, where the modulated beam passes through the PBS cube and enters the subsequent optics. A PBS with a higher contrast ratio can more effectively separate the modulated beam and significantly suppress background stray light.

The conventional PBS based on dielectric coatings operates according to the Brewster's law and the interference principle, and is highly sensitive to the angle of incidence (AOI). It maintains a high contrast ratio only within a narrow angular range, making it unsuitable for the proposed display system, which requires a wide AOI at the PBS surface to accommodate a large viewing eyebox. Otherwise, severe stray light would be introduced at peripheral viewpoints outside the central one.

Alternatively, a PBS based on wire grid polarizer, which consists of an array of parallel

metallic wires, is preferably employed in this work. It maintains a high contrast ratio over a wide AOI and broad wavelength range, making it appropriate for a large viewing eyebox and free from stray light issues. This type of PBS is commercially available from optical manufacturers such as Edmund Optics, Thorlabs, and Asahi Kasei Corporation. In this work, the selected PBS is sourced from Edmund Optics (part number: 89-604).

S3 Employing the grating vector $\boldsymbol{\Psi}_{\mathrm{G}}$ to characterize freeform HOE (FHOE)

Holographic optical element (HOE) is fabricated using the two coherent recording beams $\boldsymbol{r}_{\mathrm{S}}(x, y)$ and $\boldsymbol{r}_{\mathrm{R}}(x, y)$, where $\boldsymbol{r}_{\mathrm{S}}$ and $\boldsymbol{r}_{\mathrm{S}}$ are all defined inside the holographic medium. Assuming that the thickness of HOE is very thin, a grating vector $\boldsymbol{\Psi}_{\mathrm{G}}(x, y)$ on the 2D substrate surface can characterize HOE.

$$\boldsymbol{\Psi}_{\mathrm{G}}(x,y) = \boldsymbol{r}_{\mathrm{S}}(x,y) - \boldsymbol{r}_{\mathrm{R}}(x,y) \tag{S1}$$

Compared to spherical waves, the wavefront profile of freeform waves becomes more complex, which results in a higher degree of distribution (direction) freedom for the wave vectors $\boldsymbol{r}_{\mathrm{S}}$ and $\boldsymbol{r}_{\mathrm{R}}$ of the recording light rays on the FHOE substrate surface. This would formulate a complex distribution (normal and direction) for the grating vector $\boldsymbol{\Psi}_{\mathrm{G}}(x, y)$, thereby offering a higher ability to modulate light waves. To numerically characterize the complex distribution of vector field $\boldsymbol{\Psi}_{\mathrm{G}}(x, y)$ for FHOE, each component of $\boldsymbol{\Psi}_{\mathrm{G}}(x, y)$ can be independently described by the linear combination of the basis functions (i.e., Gaussian basis) or the polynomials (i.e., XY polynomials).

$$\begin{aligned}&\boldsymbol{\Psi}_{\mathrm{G}}(x,y) = \left[\Psi_{\mathrm{G},x}(x,y), \Psi_{\mathrm{G},y}(x,y), \Psi_{\mathrm{G},z}(x,y)\right] \\ &\text{e.g. } \Psi_{\mathrm{G},i}(x,y) = \sum_{m}\sum_{n} A^{i}_{m,n} x^{m} y^{n},\ i = \{x, y, z\}\end{aligned} \tag{S2}$$

In this case, the optimization for the imaging or display optical system based on FHOE can employ the multi-parameter optimization approach, similar to that of the conventional freeform optical system, aiming to minimize the required loss functions that relate to the system performance (e.g., point spread function, etc.), and to satisfy certain system boundary constraints by optimizing the coefficients of each term.

The light ray vector can be represented by the local gradient of wavefront profile in mathematics. After completing the optimization for the $\boldsymbol{\Psi}_{\mathrm{G}}(x, y)$, the freeform recording waves (signal wave and reference wave) required by FHOE can be obtained by inversely decomposing the vector field $\boldsymbol{\Psi}_{\mathrm{G}}(x, y)$ into the recording ray vectors $\boldsymbol{r}_{\mathrm{S}}(x, y)$ and $\boldsymbol{r}_{\mathrm{R}}(x, y)$ respectively on the FHOE surface in geometrics according to Equation (S1), where the decomposed $\boldsymbol{\Psi}_{\mathrm{G}}(x, y)$ is discretely sampled on the FHOE substrate surface. This acquisition process for the recording waves can only be implemented through the vector decomposition method, rather than the ray tracing. In the experimental setups, the required freeform waves can be generated by intentionally introducing the wavefront correction elements in the recording system, such as freeform optics, phase-only SLM, etc. According to these discrete light ray vectors $\boldsymbol{r}_{\mathrm{S}}(x, y)$ and $\boldsymbol{r}_{\mathrm{R}}(x, y)$ pre-known, the target surface or phase profile of the correction elements can generally be solved using the iterative optimization approach, by iteratively tracing the light ray vectors between the correction element and the FHOE substrate surface repeatedly.

Regarding the regulation of diffraction efficiency for FHOE, the joint optimization process is expected to be performed for both the optical (imaging or display) system based on FHOE and the holographic recording system of FHOE, which requires that these two types of systems can be optimized using the unified optimization approach. When using the grating vector $\boldsymbol{\Psi}_{\mathrm{G}}(x, y)$ to characterize the FHOE, the recording system design can only be implemented through the iterative optimization approach; however, this cannot be performed cooperatively with the multi-parameter optimization employed for the imaging or display system design based on FHOE, meaning that the joint optimization for both types of systems cannot be achieved.

To address this, the phase function $\phi_G(x, y)$ is used in this paper to numerically characterize FHOE. Both the optical system (imaging or display) based on FHOE and the holographic recording system of FHOE can all be modeled and designed through direct ray tracing, as well as optimized using the multi-parameter approach. Therefore, the joint optimization for these two types of systems can be effectively achieved, finding the optimal system solution that simultaneously considers system performance, diffraction efficiency, system constraints, as well as fabrication requirements. In addition, compared to the holographic recording system designed using the iterative optimization approach, our proposed design scheme can more effectively generate the recording waves with the higher accuracy required by FHOE, and achieve a higher system design efficiency. This is crucial for the fabrication of FHOE and the actual system performance.

S4 Constructing the loss functions to regulate the diffraction efficiency

In the proposed display system, when a display light ray $\boldsymbol{r}_\mathrm{I}$ is incident at the point P(x, y) on the fabricated FHOE surface, the attenuation of diffraction efficiency for the 1st order diffracted ray $\boldsymbol{r}_\mathrm{D}$ can be characterized by the Equation (S3).

$$\Delta\Psi_{\mathrm{G},z}(x,y)=\left|r_{\mathrm{D},z}(x,y)-\Psi_{\mathrm{G},z}(x,y)-r_{\mathrm{I},z}(x,y)\right| \tag{S3}$$

where $\Psi_{\mathrm{G},z}(x, y)$ is the z-component of the grating vector $\boldsymbol{\Psi}_\mathrm{G}(x, y)$ at this point P(x, y), as expressed in Equation (S1), which is totally determined by the design of holographic recording system for FHOE. When the parameter $\Delta\Psi_{\mathrm{G},z}(x, y)$ equals zero, fully satisfying the Bragg condition, the diffraction efficiency of the diffracted light ray $\boldsymbol{r}_\mathrm{D}$ reaches the highest.[1,2] However, deviations in the wavelength or angle of the incident wave from the recording condition of the holographic grating would result in a non-zero value for $\Delta\Psi_{\mathrm{G},z}$, leading to the attenuation of diffraction efficiency. The data in Equation (S3) are all associated with both the holographic display system and the holographic recording system, and can be exactly obtained through direct ray tracing. Therefore, employing the parameter $\Delta\Psi_{\mathrm{G},z}$ to regulate the diffraction efficiency of the light rays diffracted by the FHOE used as the OC requires the joint optimization for these two types of systems. To implement this, the loss function related to regulating diffraction efficiency needs to be constructed numerically.

In this research, the aperture stop of the proposed display system is set at the LCoS plane, thereby fully utilizing the entire active area of the LCoS panel. This means that the entire bundle of light rays for any viewpoints fully cover the active aperture of the LCoS panel. To regulate the diffraction efficiency of any specific display rays for any sampled viewpoint E_i, the traced feature light rays must be sampled across the entire aperture stop range (i.e., the LCoS aperture). For the simplicity, the parameter $\Delta\Psi_{\mathrm{G},z}$ corresponding to the k-th sampled light ray across the aperture stop for the sampled viewpoint E_i is denoted as $\Delta_{i,k}$.

To achieve uniform display brightness across the viewing field-of-view (FOV) range, the uniformity of diffraction efficiency among the sampled light rays for the viewpoint E_i should be regulated, which is implemented by minimizing the standard deviation (SD) of all the sampled parameters $\Delta_{i,k}$.

$$\mu_i=\sqrt{\frac{\sum_{k}^{K}\left(\Delta_{i,k}-\frac{\sum_{j}^{K}\Delta_{i,j}}{K}\right)^2}{K}} \tag{S4}$$

where K is the total number of the sampled light rays across the aperture stop. The loss function used to regulate the uniformity of diffraction efficiency for the viewpoint E_i is formulated based on the parameter μ_i.

$$\mathcal{L}_{\mathrm{SD},i} = w_{\mathrm{SD},i}(\mu_i - \mu_{\mathrm{target},i})^2 \tag{S5}$$

where $w_{\mathrm{SD},i}$ is the weight value, and $\mu_{\mathrm{target},i}$ is the target value, which is set to zero during the optimization.

For the sampled viewpoint E_i, the root-mean-square (RMS) value of the parameters $\Delta_{i,k}$ for all the sampled light rays can represent the magnitude of diffraction efficiency for this viewpoint.

$$\eta_i = \sqrt{\frac{\sum_k^K \Delta_{i,k}^2}{K}} \tag{S6}$$

Similarly, the loss function used to regulate the magnitude of diffraction efficiency for the sampled viewpoint E_i is represented as follows.

$$\mathcal{L}_{\mathrm{RMS},i} = w_{\mathrm{RMS},i}(\eta_i - \eta_{\mathrm{target},i})^2 \tag{S7}$$

where $w_{\mathrm{RMS},i}$ is the weight, and $\eta_{\mathrm{target},i}$ is the target value.

In addition, to regulate the uniformity of diffraction efficiency among the sampled viewpoints, the standard deviation of the parameter η_i for all the sampled viewpoints E_i is computed, thereby achieving uniform display brightness across the viewing eyebox range.

$$\mu = \sqrt{\frac{\sum_i^I \left(\eta_i - \frac{\sum_j^I \eta_j}{I}\right)^2}{I}} \tag{S8}$$

where I is the total number of the sampled viewpoints. The corresponding loss function is represented as follows.

$$\mathcal{L}_{\mathrm{SD}} = w(\mu - \mu_{\mathrm{target}})^2 \tag{S9}$$

where w is the weight value, and μ_{target} is the optimization target value.

As described above, the loss function related to regulating the diffraction efficiency (DE) can be formulated as follows.

$$\mathcal{L}_{\mathrm{DE}} = \sum_i^I \mathcal{L}_{\mathrm{SD},i} + \sum_i^I \mathcal{L}_{\mathrm{RMS},i} + \mathcal{L}_{\mathrm{SD}} \tag{S10}$$

In this paper, the total loss function $\mathcal{L}_{\mathrm{total}}$ employed for the joint optimization comprises the loss function $\mathcal{L}_{\mathrm{DE}}$ related to diffraction efficiency shown in Equation (S10), as well as the loss functions $\mathcal{L}_{\mathrm{display}}$ and $\mathcal{L}_{\mathrm{recording}}$ related to the system performance (e.g., spot size) for both the holographic display system and the holographic recording system. The joint optimization target is essentially to find the solution that minimizes the total loss function, and satisfies the certain boundary constraints (e.g., avoiding structural interference, lights obscuration, stray lights etc.). This process can be implemented in a least-square sense through the multi-parameter optimization approach.

S5 The construction process for the proposed display system

The design of the proposed display system is modeled in a reverse manner (i.e., tracing the light rays from the viewpoints to the illumination points), as shown in **Figure S1**(a). The design target is to achieve the less-aberration mapping between the viewpoints and the illumination points, that is to say, a spherical wave emitted from a viewpoint can map to the quasi-spherical wave converging at an illumination point (within a single pixel unit of illumination source).

The modulation of LCoS on the illumination beams is directly modeled using a planar reflective surface, while considering the cover glass during modeling, owing to the fact that the liquid crystal (LC) layer is fairly thin.

The proposed display system is constructed symmetrically about the *yoz* plane, with a global reference coordinate system located at the center of eyebox. To fully utilize the active area of LCoS panel, and effectively eliminate the stray lights, the aperture stop of this display scheme is positioned at the LCoS plane. For the viewpoint E_c located at the center across the expanded eyebox, the optical combiner (OC) deflects and focuses the divergent spherical wave emitted from E_c into a point P_c serving as an intermediate image point, thereby enabling an AR see-through viewing, as well as ensuring a compact system form factor. Then, a lens L1 collimates the spherical wave from the point P_c to ensure the uniform modulation by the LCoS panel. Correspondingly, for the illumination unit of LCoS, a collimation lens L2 generates a spherical wave converging at the illumination point I_c, eventually achieving a less-aberration mapping between points E_c and I_c. Considering that other viewpoints E_i are distributed symmetrically around the viewpoint E_c, the light rays corresponding to the viewpoint E_c are set to be incident perpendicularly on the LCoS panel.

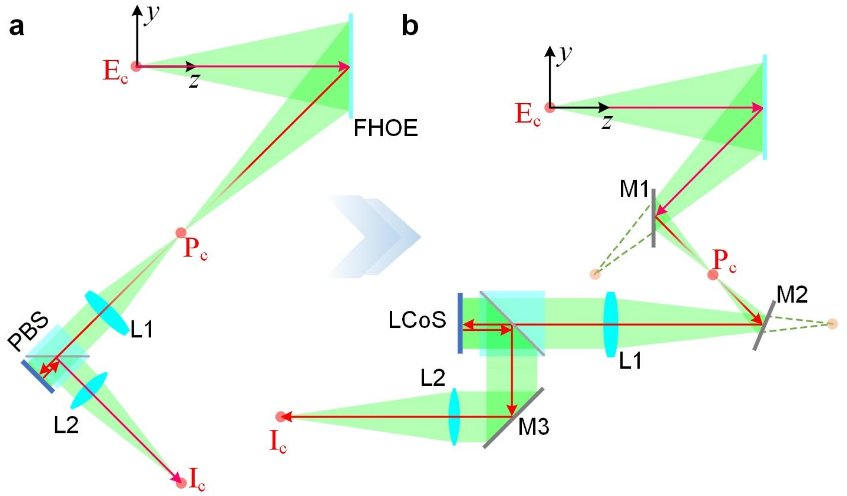

Figure S1. Construction process of the proposed display system. a) Constructed system structure. b) A more compact structure by folding the light paths through mirrors.

However, the system structure shown in Figure S1(a) is too large and has low applicability. To achieve a more compact form factor, several mirrors M1~M3 are utilized to fold the light paths, as shown in Figure S1(b). Considering aesthetics, conciseness, and ease to assembly, OC is set perpendicular to the visual axis of human eye without any tilt, that is, perpendicular to the global *z*-axis, and LCoS plane is positioned parallel to the global *y*-axis. Owing to the increased optical path length achieved by folding the lights, the optical powers required for the OC and the collimation lens L1 can be further reduced, which is conducive to modulating the light waves (correcting aberrations) and reducing the design difficulty.

S6 The boundary constraints for the display system and the holographic recording system

S6.1 Display system

During the optimization, several boundary constraints must be constructed to ensure a feasible system structure and desired display requirements.

1) Constrain the spacing between each component, as indicated by the double-arrow red lines in **Figure S2**, to avoid structure interference and light obscurations, ensuring that the off-axis structure does not interfere with the head or occlude the eye's viewing, and to achieve a compact system form factor.

2) Constrain the incidence angle of the light rays on the LCoS panel with a limited range (≤ 12 °) to avoid the pixels crosstalk. According to the imaging principles, for the viewpoints E_i located at the edge of eye pupil plane, the corresponding incidence angle on the LCoS would reach the maximum. Therefore, the incidence angles α of the light rays from these edge viewpoints on the LCoS surface are constrained to be less than 12 °, as shown in Figure S2.
3) A telecentric light path is required for the eye pupil plane and the illumination source plane, achieving an acceptable viewing way for the human eye, and accurately simulating the emission characteristics of the actual illumination source (i.e., micro-OLED).

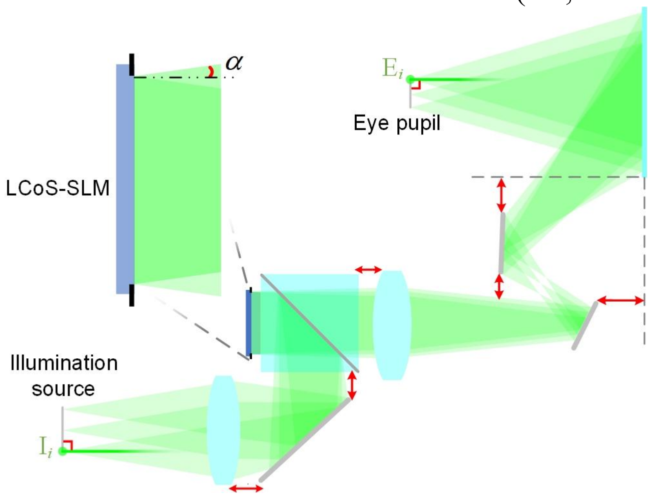

Figure S2. Boundary constraints for the display system.

S6.2 Holographic recording system

For the design of holographic recording system, the boundary constraints mainly consider avoiding the structure interference, light obscurations, and stray lights, such as constraining the spacing between components, as indicated by the double-arrow red lines in **Figure S3**(a). In addition, the telecentric light path is required for both the recording point sources P_S and P_R, accurately simulating the emission characteristics of the actual spherical waves from the microscope objective. Regarding the stray lights, constraining the orientation of the freeform mirror ensures that the light rays transmitted directly through the HOE and the substrate layer are not reflected back to the HOE substrate surface, as indicated by the purple rays in Figure S3(b). Otherwise, stray lights would be induced during the recording process of the holographic grating, which deteriorates display performance.

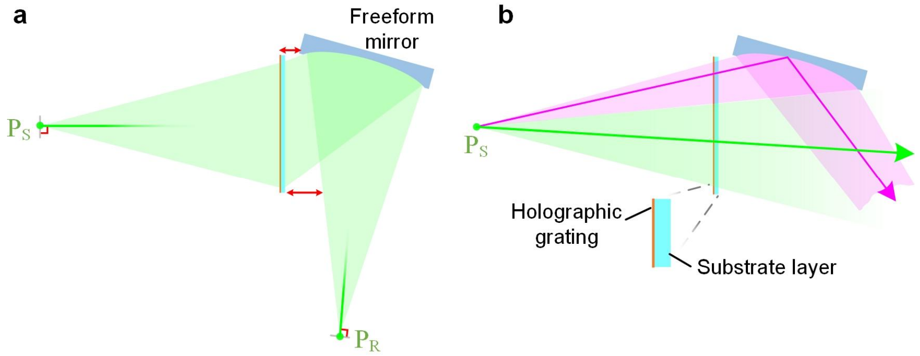

Figure S3. Boundary constraints for the holographic recording system. a) Boundary constraint. b**)** Constraint on stray lights.

S7 Design results

After joint optimization, the layout of the display system is shown in **Figure S4**, where the light rays of different colors indicate the viewpoints located at the different positions across the eye

pupil plane. The ray deflections caused by the cover glass protecting the holographic grating are considered during the design.

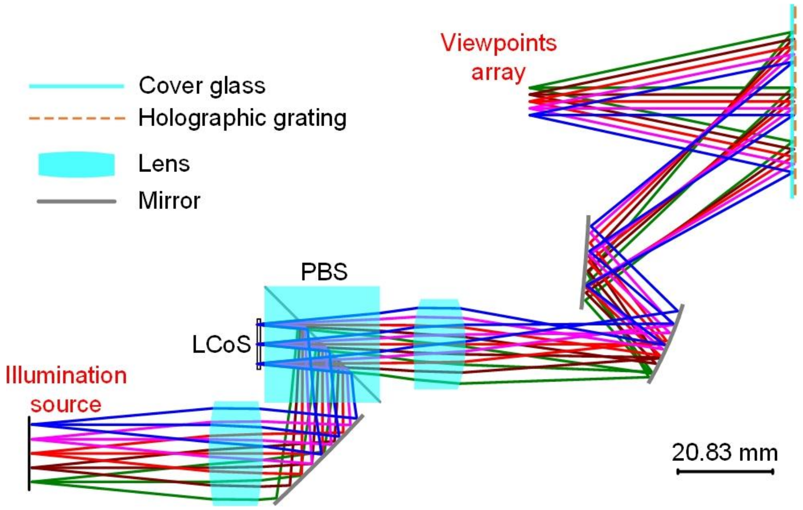

Figure S4. Layout of the designed display system.

The system layout, which positions a perfect lens at the LCoS plane, is shown in **Figure S5**(a). This design aims to ensure that the illumination waves incident on the LCoS panel are quasi-planar, thereby achieving uniform modulation. As shown in Figure S5(b), corresponding to the sampled viewpoints array across the eye pupil plane, the RMS spot size of the 'virtual' points formed by the perfect lens become relatively small (less than 0.2 mm), meaning that the illumination waves on the LCoS panel are nearly quasi-planar waves.

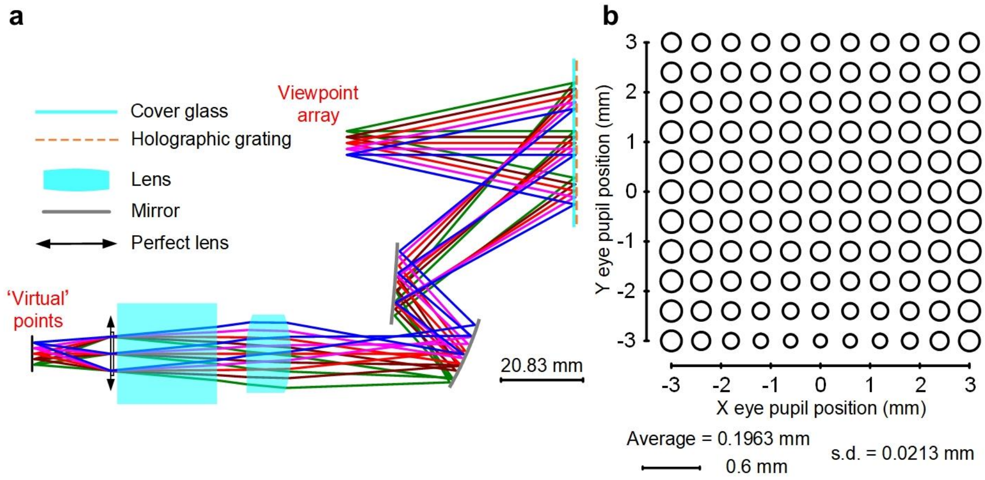

Figure S5. Evaluation of the collimation degree for the illumination waves incident on the LCoS panel. a) System layout that positions a perfect lens at the LCoS plane. b) RMS spot size of the 'virtual' points corresponding to the sampled viewpoints array.

The layout of the holographic recording system for FHOE is shown in **Figure S6**(a). The aperture stop is positioned at the FHOE surface, and its size is slightly larger than the active area of the FHOE applied to the display system. This ensures that the recording beams can fully cover the required grating area. In addition, the ray deflections caused by the substrate glass supporting the holographic grating are also considered for the design of the recording system. The spot size of the optimized point source, as shown in Figure S6(b), is sufficiently small (less than 10 μm). In the experimental setup, the quasi-spherical waves emitted from these two points sources, after being modulated by the freeform mirror, can reconstruct the required recording waves on the FHOE surface.

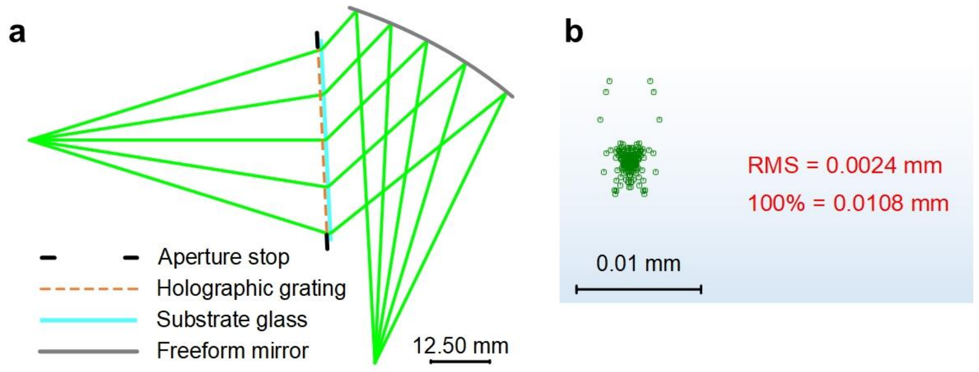

Figure S6. Holographic recording system. a) Layout of the holographic recording system for FHOE. b) Spot size of the optimized point source.

S8 Diffraction efficiency of freeform HOE

After exposure to coherent waves, the refractive index distribution of the holographic medium is modulated by the interference pattern formed by the signal wave and reference wave. The spatial modulation of the refractive index within a volume grating structure can be expressed as follows. [3,4]

$$n(\boldsymbol{r}) = n + \Delta n \cos(\boldsymbol{\Psi}_G \cdot \boldsymbol{r}) \tag{S11}$$

where Δn is the amplitude of refractive index modulation, and n is the average refractive index of the holographic medium. $\boldsymbol{r}$ is the radial vector defined as $\boldsymbol{r}=(x, y, z)$.

Based on the coupled wave theory (CWT), the diffraction efficiency can be calculated by solving the coupled wave equations with boundary conditions in a volume grating structure.[3] This theory is a first-order two-wave theory, assuming that there is only 0th and 1st order diffracted wave in the grating region, and the other diffraction orders are neglected. The diffraction efficiency η for a reflection volume holographic grating can be computed by the Equation (S12).[5]

$$\eta = \kappa^2 \frac{\sinh(\gamma d)^2}{\gamma^2 \cosh(\gamma d)^2 + (\frac{\Delta \Psi_{G,z}}{2})^2 \sinh(\gamma d)^2} \tag{S12}$$

where $\Delta \Psi_{G,z}$ is the phase mismatch term, defined in Equation (S3), and d is the thickness of holographic medium.

$$\kappa = \frac{2\pi \Delta n}{\lambda_p \|\boldsymbol{\Psi}_G\|} \frac{2\pi n_c}{\lambda_c} \tag{S13}$$

$$\gamma^2 = \kappa^2 - (\frac{\Delta \Psi_{G,z}}{2})^2 \tag{S14}$$

where λ_c is the wavelength of the recording waves, and n_c is the refractive index of the holographic medium. λ_p is the wavelength of the reconstruction waves. $\boldsymbol{\Psi}_G$ is the grating vector defined by the Equation (S1), and it can be further expanded as follows. Equation (S13) represents the coupling constant of volume holographic grating.

$$\boldsymbol{\Psi}_G = \boldsymbol{r}_S - \boldsymbol{r}_R = \frac{2\pi n_c}{\lambda_c}(\mu_S - \mu_R, \nu_S - \nu_R, \xi_S - \xi_R) \tag{S15}$$

where μ, ν, ξ are the direction cosine relative to the local x, y, z axis of substrate surface. The subscripts ‘S’ and ‘R’ denote the recording signal and the reference waves, respectively.

According to Equation (S15), the Equation (S13) can be deduced as follows:

$$\kappa = \frac{2\pi\Delta n}{\lambda_P\sqrt{(\mu_S-\mu_R)^2+(\nu_S-\nu_R)^2+(\xi_S-\xi_R)^2}} \tag{S16}$$

Additionally, the phase mismatch $\Delta\Psi_{G,z}$ can also be further expressed as follows:

$$\Delta\Psi_{G,z}(x,y) = \left|\frac{2\pi n_P}{\lambda_P}\xi_D - \frac{2\pi n_C}{\lambda_C}(\xi_S-\xi_R) - \frac{2\pi n_P}{\lambda_P}\xi_I\right| \tag{S17}$$

where the subscripts 'D' and 'I' denote the diffracted and incident waves, respectively, for the reconstruction process. The data in Equation (S13) and Equation (S14) can all be obtained through direct ray tracing and from the photopolymer manufacturer. Employing the Equation (S12) can quantitatively evaluate the distribution of diffraction efficiency η for the proposed holographic display system in this work.

As described in Section S4, the optimization of diffraction efficiency aims to regulate both the magnitude and uniformity across the viewing eyebox and FOV range. For the nine sampled viewpoint position E_i within the eyebox range (as marked by the nine colored-point in **Figure S7**), the distribution of diffraction efficiency for the traced feature rays across the entire pupil aperture range (i.e., the entire FOV range) is shown in Figure S7, where the total number of feature rays is 13398 for each viewpoint.

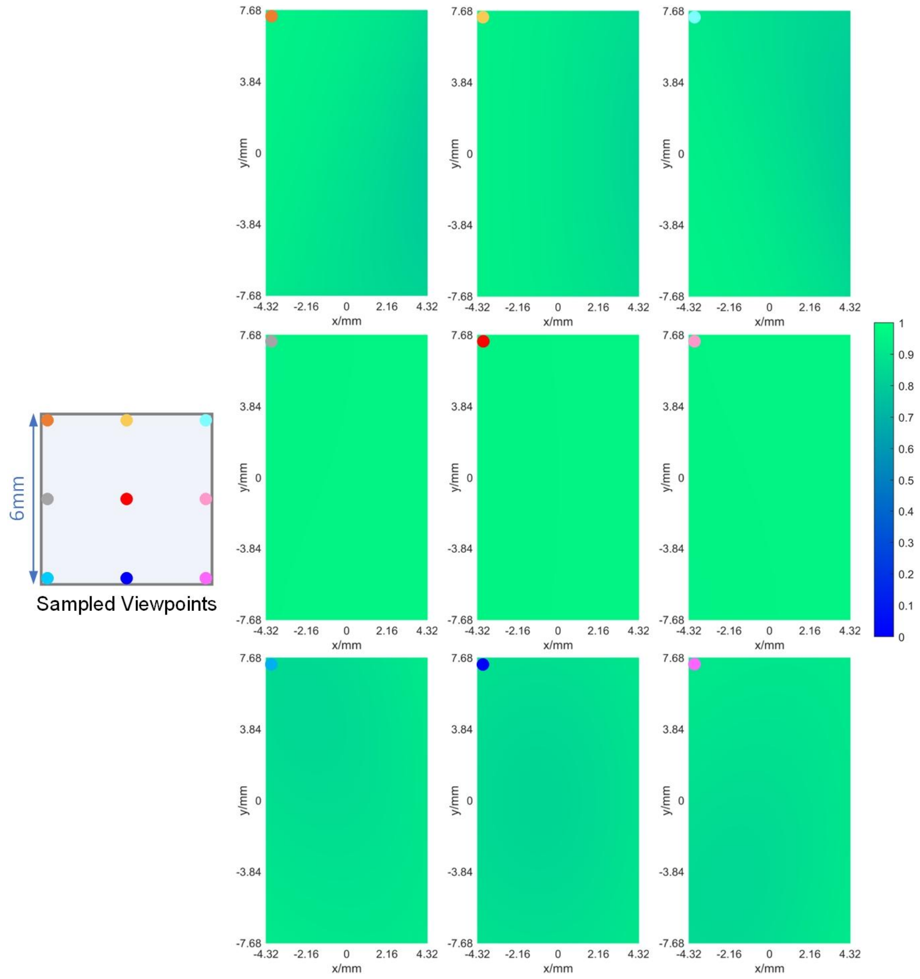

Figure S7. The distribution of diffraction efficiency for the entire pupil aperture range at nine sampled viewpoints within eyebox range.

Then, by sampling viewpoints within the viewing eyebox range of 6mm×6mm at 0.1mm intervals, the RMS and SD values of the diffraction efficiency of all sampled feature rays for each viewpoint are presented in **Figure S8**(a) and Figure S8(b), respectively. The total number

of feature rays for each viewpoint remains 13398. As shown in Figure S8(a), the diffraction efficiency at all sampled viewpoints exceeds 85%, and the SD value of the diffraction efficiency is 0.023, which enables high and uniform display brightness across the entire viewing eyebox range. Additionally, as shown in Figure S8(b), for each sampled viewpoint, the SD value of the diffraction efficiency of all sampled feature rays is less than 0.03, ensuring uniform display brightness across the entire FOV range.

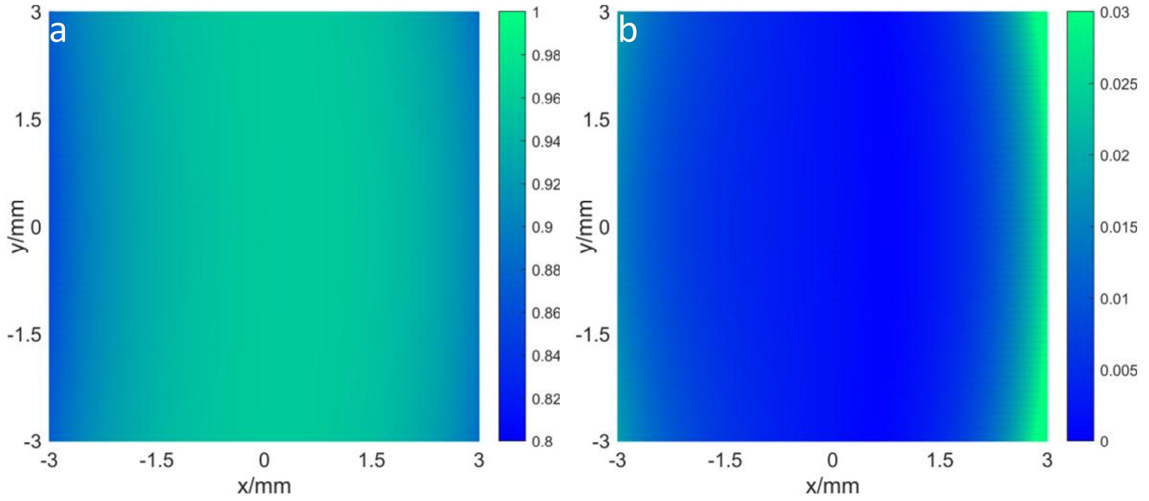

Figure S8. a) RMS values of the diffraction efficiency of all sampled feature rays at each sampled viewpoint across the entire viewing eyebox range. b) SD values of the diffraction efficiency of all sampled feature rays at each sampled viewpoint across the entire viewing eyebox range.

S9 Display results

At the different viewpoint positions E_i ($1 \leq i \leq 9$), as the camera changes the focus from 0.25 m to 10 m, the captured virtual images are shown in following figures.

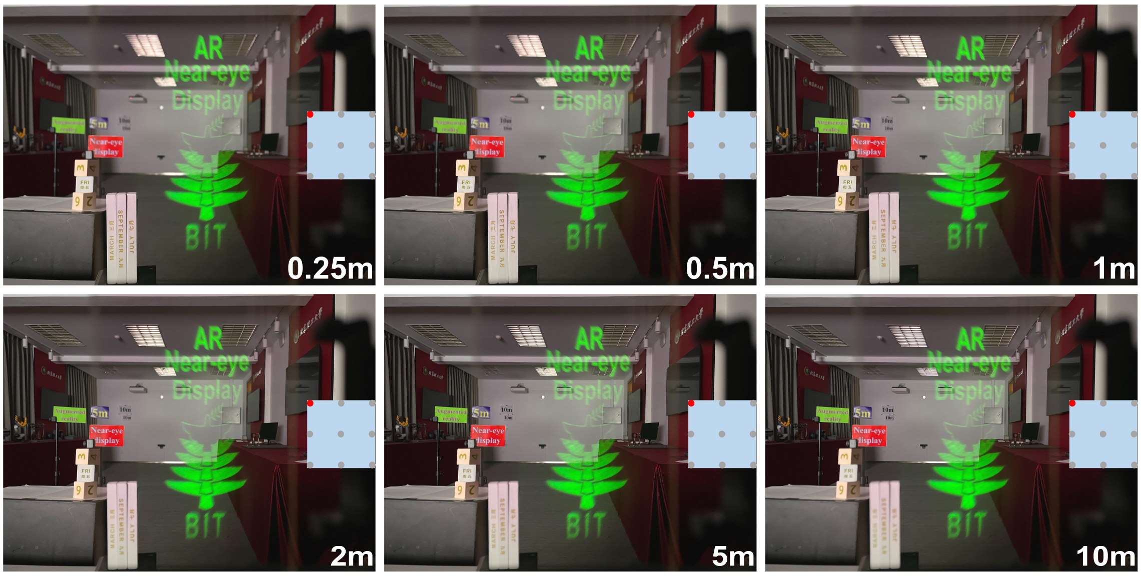

Figure S9. Viewpoint position E_1.

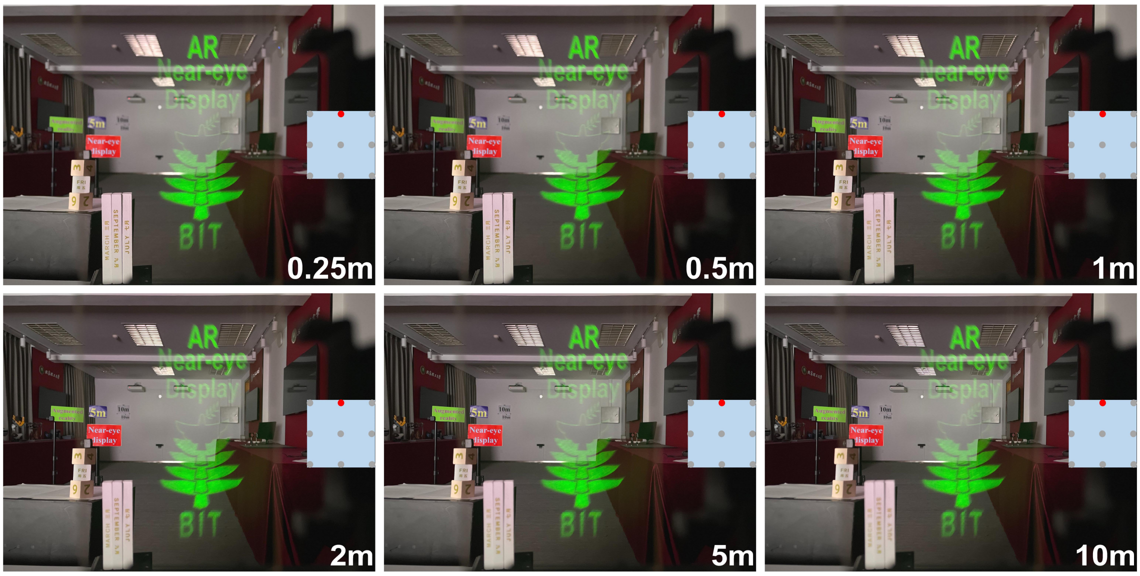

Figure S10. Viewpoint position E_2.

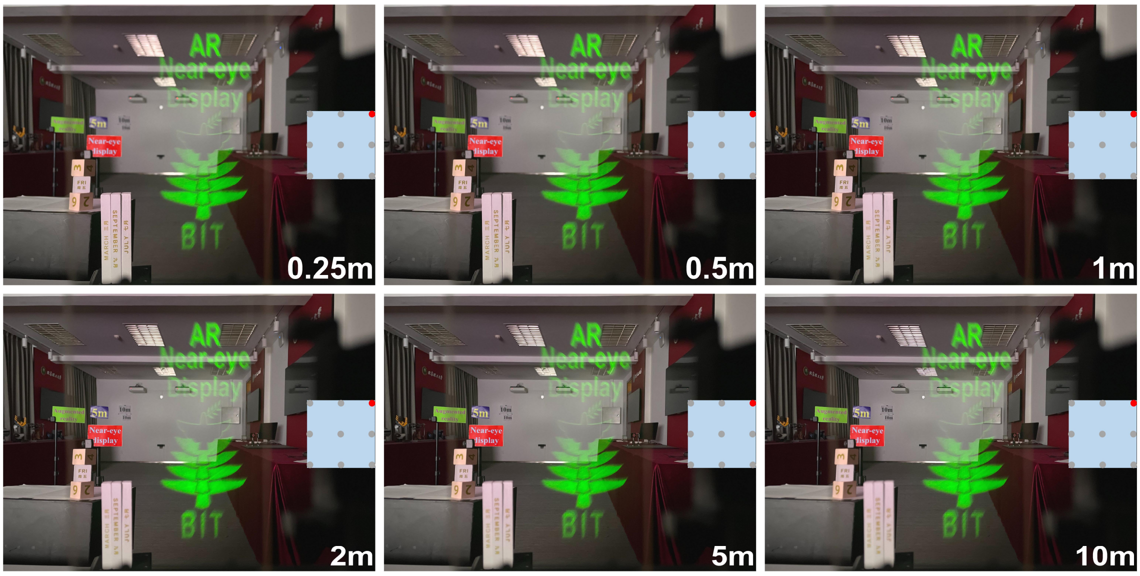

Figure S11. Viewpoint position E_3.

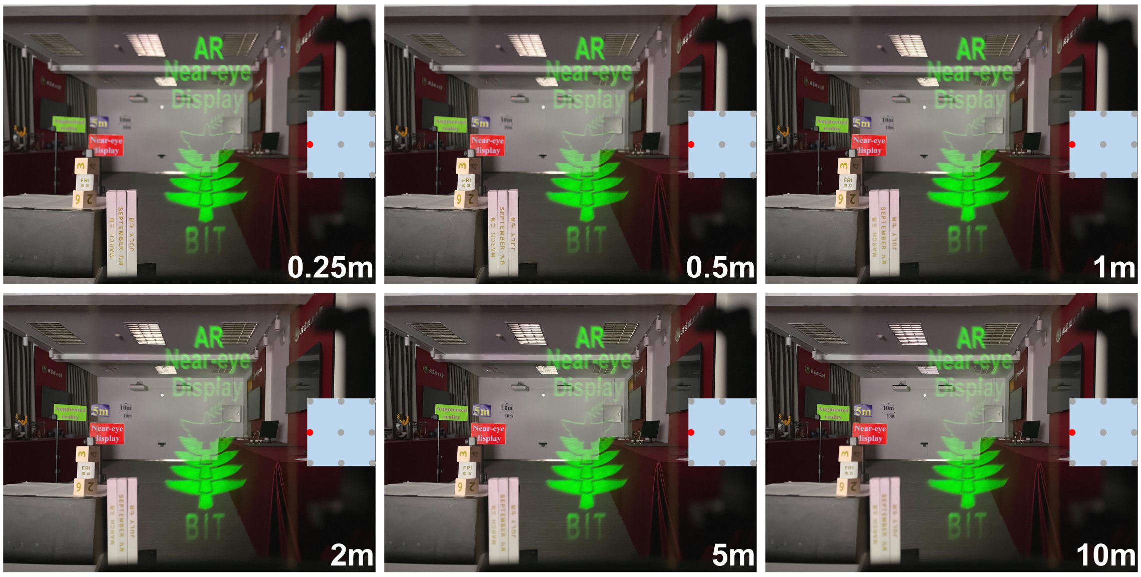

Figure S12. Viewpoint position E_4.

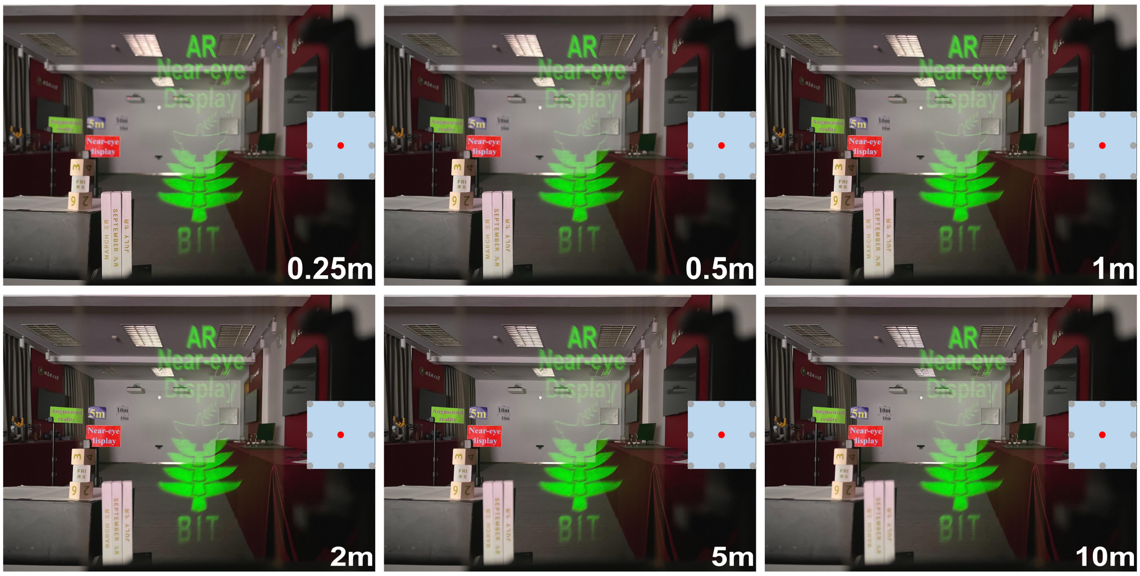

Figure S13. Viewpoint position E_5.

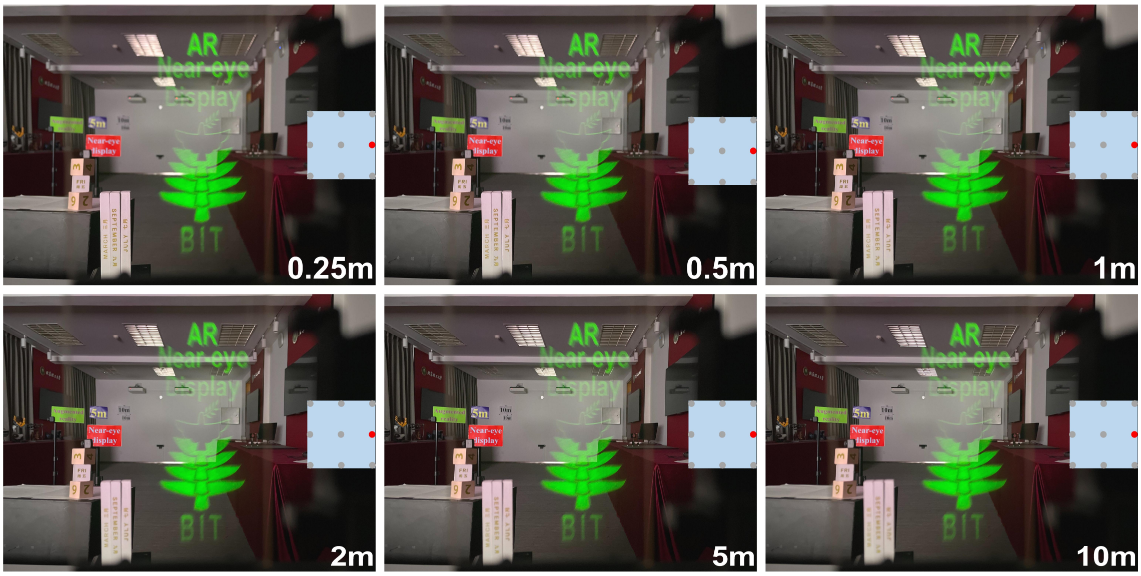

Figure S14. Viewpoint position E_6.

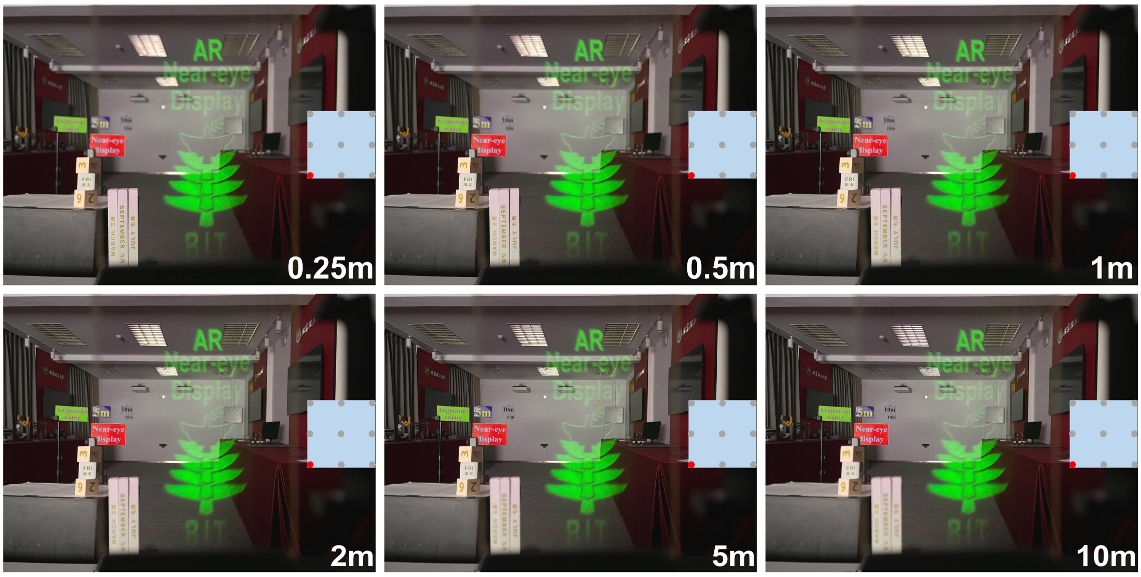

Figure S15. Viewpoint position E_7.

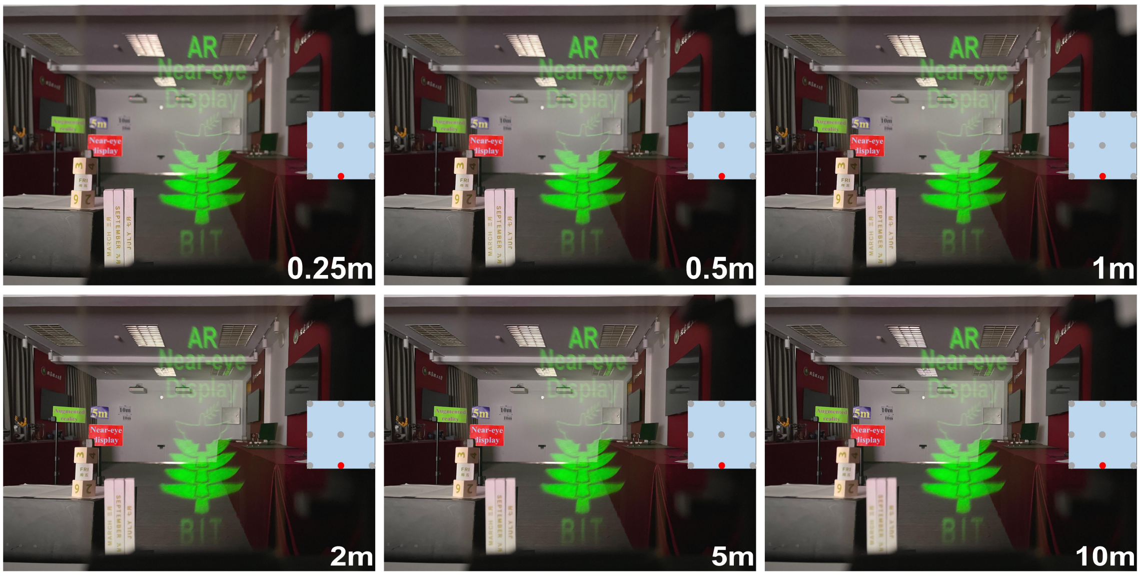

Figure S16. Viewpoint position E_8.

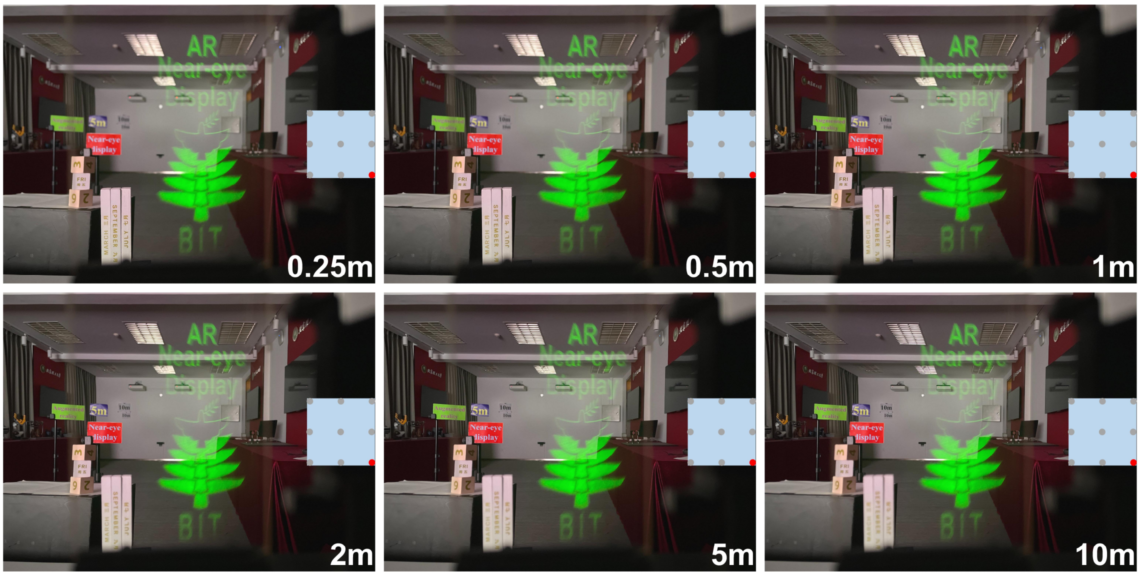

Figure S17. Viewpoint position E_9.

References

[1]. D. Psaltis, M. Levene, A. Pu, G. Barbastathis, K. Curtis, *Opt. Lett.* **1995**, 20, 782.
[2]. P. Wissmann, S. Oh, G. Barbastathis, *Opt. Express* **2008**, 16, 7516.
[3]. H. Kogelnik, *Bell Syst. Tech. J.* **1969**, 48, 2909.
[4]. C. Jang, O. Mercier, K. Bang, G. Li, Y. Zhao, D. Lanman, *ACM Trans. Graph.* **2020**, 39, 1.
[5]. Yariv, P. Yeh, Optical waves in crystals: propagation and control of laser radiation, *Wiley Classics Library*, Wiley (New York), **2003**. ISBN: 9780471430814.
